# Ion Channels in Gliomas—From Molecular Basis to Treatment

**DOI:** 10.3390/ijms24032530

**Published:** 2023-01-28

**Authors:** Abdallah F. Elias, Bernice C. Lin, Beverly J. Piggott

**Affiliations:** 1 Department of Medical Genetics, Shodair Children’s Hospital, Helena, MT 59601, USA; 2 Division of Biological Sciences, University of Montana, Missoula, MT 59812, USA; 3 Division of Medical Genetics, Department of Pediatrics, University of Utah, Salt Lake City, UT 84112, USA; 4 Center for Biomolecular Structure and Dynamics, University of Montana, Missoula, MT 59812, USA; 5 Center for Structural and Functional Neuroscience, University of Montana, Missoula, MT 59812, USA

**Keywords:** ion channels, ion exchanger, ion transporter, brain cancer, glioma, glioblastoma, tumor microenvironment, drug target, blood–brain barrier, tumor-associated epilepsy, angiogenesis, treatment resistance

## Abstract

Ion channels provide the basis for the nervous system’s intrinsic electrical activity. Neuronal excitability is a characteristic property of neurons and is critical for all functions of the nervous system. Glia cells fulfill essential supportive roles, but unlike neurons, they also retain the ability to divide. This can lead to uncontrolled growth and the formation of gliomas. Ion channels are involved in the unique biology of gliomas pertaining to peritumoral pathology and seizures, diffuse invasion, and treatment resistance. The emerging picture shows ion channels in the brain at the crossroads of neurophysiology and fundamental pathophysiological processes of specific cancer behaviors as reflected by uncontrolled proliferation, infiltration, resistance to apoptosis, metabolism, and angiogenesis. Ion channels are highly druggable, making them an enticing therapeutic target. Targeting ion channels in difficult-to-treat brain tumors such as gliomas requires an understanding of their extremely heterogenous tumor microenvironment and highly diverse molecular profiles, both representing major causes of recurrence and treatment resistance. In this review, we survey the current knowledge on ion channels with oncogenic behavior within the heterogeneous group of gliomas, review ion channel gene expression as genomic biomarkers for glioma prognosis and provide an update on therapeutic perspectives for repurposed and novel ion channel inhibitors and electrotherapy.

## 1. Introduction—Classification and Etiopathogenesis of Brain Tumors

Tumors of the central nervous system (CNS) are devastating diseases ranging among the top challenges in healthcare due to their high morbidity and mortality in all ages. They are the seventh most common neoplasm in adults and, representing 25% of childhood malignancies, are the second leading cause of cancer death in children and young adults [1,2]. Understanding the role of ion channels in the pathogenesis of brain tumors requires an understanding of the type of brain cell and the genetic context from which they originate. It is also increasingly important to have a suitable understanding of CNS tumor classification. An authoritative reference for the classification of brain neoplasms, the 2021 “WHO Classification of Tumours, 5th Edition, Volume 6: Central Nervous System Tumours” expands on major advances of molecular diagnostics that were first introduced in the 2016 updated fourth edition to aid CNS tumor taxonomy [3]. These developments should be considered when appraising current research on ion channels in CNS tumors as they allow for greater standardization of tumor backgrounds in research studies, thereby providing context for broader translational efforts in this field. Primary brain tumors develop in brain cells and are classified by the type of tissue in which they arise. Although the principal functional unit of the CNS is the neuron, most primary brain tumors are named according to other brain cells, such as astrocytes and oligodendrocytes, which represent subtypes of glial cells. The major classes of primary brain tumors include gliomas, neuronal/glioneuronal tumors, embryonal tumors, and a few rarer types. The most common group of primary brain tumors are the gliomas encompassing astrocytomas, oligodendrogliomas, and ependymomas. Although the primary basis for the classification of these tumor types remains their characteristic histology, these tumors are derived from multipotent progenitor cells that preferentially differentiate along a particular cellular lineage, and molecular genetic characterization has moved to the forefront of diagnostic, management, and prognostic considerations.

Astrocytic tumors are divided into diffusely infiltrating astrocytomas (WHO grade II to IV) and more localized astrocytomas, most commonly pilocytic astrocytomas (WHO grade I). Astrocytomas may occur from the first decade of life onward with diffusely infiltrating astrocytoma and glioblastoma, also referred to as grade IV astrocytoma, accounting for 80% of primary brain tumors in adults. In older individuals, glioblastoma typically occurs as a new-onset disease (primary glioblastoma), while in younger patients, it arises as a result of progression from a lower-grade astrocytoma (secondary glioblastoma). Secondary glioblastomas and their lower-grade precursors are frequently associated with driver mutations of *IDH1*. In contrast, primary glioblastomas are typically *IDH* wild type, featuring instead other types of genomic variation, including copy number gains of chromosome 7, copy number loss of chromosome 10, *TERT* (telomerase reverse transcriptase) promoter mutations, and *EGFR* (epidermal growth factor receptor) gene amplification. This genomic instability lays the groundwork for cancer transformation and malignant glioblastoma behavior, which is characterized by evasion of senescence (telomerase or telomere length affecting mutations), escape from growth and invasion control (biallelic *CDKN2A* deletion), activation of growth factor signaling pathways (*EGFR* or *PDGFR* (platelet-derived growth factor receptor) gene amplification), and resistance to programmed cell death (*TP53* mutation).

As cancer progresses, alterations of other genes and their biological pathways are likely to promote more aggressive cancer phenotypes. Genes encoding ion channels in gliomas are differentially expressed compared with unaffected brain tissue and are thought to drive aspects of brain cancer development and potentially influence patient survival rates (Table 1) [4]. Today, there exists a plethora of studies underscoring the relevance of ion channels for each cancer hallmark associated with glioblastoma, including self-sufficiency in growth signals, insensitivity to antigrowth signals, evasion of apoptosis, limitless replicative potential, sustained angiogenesis, and tissue invasion and infiltration [5]. Although much of what we know about ion channels in brain tumors is derived from work in diffuse infiltrating astrocytomas, and here, especially the aggressive glioblastoma, similar considerations apply to other types of brain tumors and will be discussed in this review where applicable and relevant. The other major subtype of infiltrating glioma is comprised of cells that resemble oligodendrocytes. These tumors typically are diagnosed in the fourth and fifth decades and have the most favorable prognosis among glial tumors. Molecularly, oligodendrogliomas are *IDH* mutation-positive and feature deletions of chromosomes 1p and 19q. Progression to anaplastic oligodendroglioma is associated with additional genetic changes, such as loss of chromosome 9p, resulting in deletion of the *CDKN2A* tumor suppressor gene and the same *TERT* promoter mutations detected in glioblastoma [6]. Ependymomas, the last subtype of gliomas mentioned here, arise in the internal lining of the brain’s ventricles and the spinal cord’s central canal. They are relatively common in the first two decades of life, constituting 5% to 10% of primary brain tumors and typically occur near the fourth ventricle [6]. In contrast, adults tend to develop ependymomas in the spinal cord, a location that is more frequently encountered in individuals with underlying neurofibromatosis type 2 (NF2). Non-glial tumors to be briefly mentioned include choroid plexus tumors, neuronal or glioneuronal tumors, and embryonal tumors. Choroid plexus tumors are intraventricular tumors that most commonly occur in children presenting with hydrocephalus due to tumor obstruction of the ventricular system or overproduction of cerebrospinal fluid (CSF). Tumors with neuronal differentiation, particularly gangliogliomas (WHO grade I) are relatively rare and more frequently observed in children and adolescents presenting with seizures. Up to 50% may harbor the common activating V600E mutation in the *BRAF* gene, although more recent studies have highlighted genetic alterations that activate the MAP kinase pathway [7]. The embryonal tumors represent a large class of primarily pediatric tumors. This group of neoplasms derives its name from their undifferentiated appearance. The most common embryonal tumor is the medulloblastoma, which occurs exclusively in the cerebellum and accounts for 20% of pediatric brain tumors. Medulloblastomas show alterations in the sonic hedgehog-patched (SHH) and the WNT/β-catenin signaling pathways, both involved in cerebellar development. The molecular signature provides the basis for classification of medulloblastoma subtypes, which in turn informs prognosis, with WNT-activated medulloblastomas being associated with a 5-year survival of almost 100%, which drops to 20% to 30% for more aggressive subtypes [8].

Tumor behavior in primary CNS malignancies differs from the neoplasm of other tissues in several aspects. For example, diffuse infiltrating gliomas tend to spread widely throughout the brain parenchyma, precluding complete surgical resection without compromising neurologic function. Further, even malignant gliomas rarely metastasize outside the CNS but may spread through the cerebrospinal fluid, resulting in cerebrospinal dissemination far from the original tumor site, as can be seen with some pediatric tumors. The study of ion channels in the pathogenesis of brain tumors also needs to consider the complex interaction of oncogenic programs intrinsic to the tumor and the microenvironmental context for both neurons and glial cells. This is particularly true for glioblastoma, by far the most frequent tumor background in which ion channels have been studied. As mentioned above, glioblastoma is the most aggressive brain tumor whose poor prognosis and therapeutic resistance are rooted in glioblastoma stem cells (GSC), conferring broad cellular heterogeneity and environmental adaptability. Intratumoral heterogeneity has been implicated in aggressive behavior, treatment resistance, and poor prognosis of glioblastoma. On a cellular level, a plurality of cellular states resembling neuronal precursor cells (NPCs), oligodendrocyte precursor cells (OPCs), astrocytes, and mesenchymal cells has been described [40,41]. In addition, recent seminal studies by Venkataramani and colleagues combining sophisticated 3D in vivo imaging techniques with single-cell RNA (scRNA) sequencing have started to provide models in which a subset of malignant glioblastoma cells (GBCs) can be classified transcriptionally and phenotypically into cellular states that recapitulate certain features of neuronal and neural-progenitor-like cells. The analogies include similarities to NPC-like migration behavior and neuron-GBC synaptic activity driving GBM invasion and progression. The authors also demonstrated how plasticity between these cell types can drive tumor heterogeneity, and observed interaction of tumor cells with the tumor microenvironment, setting the stage to better model these important determinants of treatment outcome and prognosis [40,41,42]. Moreover, the characteristic diffuse invasion and evasion from therapeutic interventions appear to be driven by subpopulations of neuronal and neural-progenitor-like tumor cells in a process resembling developmental neuronal migration that is anatomically compartmentalized to the tumor rim where Lévy-like movement environmental probing patterns are observed. These recent developments have important implications for the study of ion channels in glioblastoma. For one, they provide an experimental framework in which their pathophysiological function can be studied and pharmacologically targeted within a modeled tumor microenvironment for the development of novel therapies. As neuronal activity is linked to the invasion of glioblastoma cells and associated with complex dynamics of calcium shifts, it provides support to more broadly study ion channels in these models. Furthermore, the demonstration that neuronal synaptic input to glioblastoma cells appears to be mediated via alpha-amino-3-hydroxy-5-methyl-4-isoxazolepropionic acid receptor-type glutamate receptors (AMPARs) that are enriched in glioblastoma cells exhibiting neuronal-like and neurodevelopmental cell states, but not mesenchymal states, emphasizes the importance of the environmental and cellular context in which ion channels are studied [41,42].

The major focus of this review centers on the role of ion channels in glioblastoma as the vast majority of data have been and continue to be produced for this most common and aggressive primary brain tumor in adults. Clearly, the poor prognosis of glioblastoma and the relative ineffectiveness of current treatment approaches call for new and more efficacious therapeutic targets to improve patient outcomes. However, broadening the scope of ion channel research to include other brain tumor types mentioned in our introduction may provide insights into common pathomechanisms of ion channel behaviors and benefit future management of pediatric tumors or tumor-associated epilepsy (TAE), for example. In this review we discuss our current understanding of ion channels as it pertains to the distinct yet related cell behaviors including proliferation, infiltration, and metabolism. In addition, we explore related research focused on ion channel functions in the context of the glioma microenvironment, tumor-related epilepsy, and angiogenesis—emerging fields that highlight possible roles of ion channels in important determinants of treatment response and prognosis. Finally, we discuss the potential for ion channel therapies as a treatment for gliomas.

## 2. Proliferation

**Figure 1:**
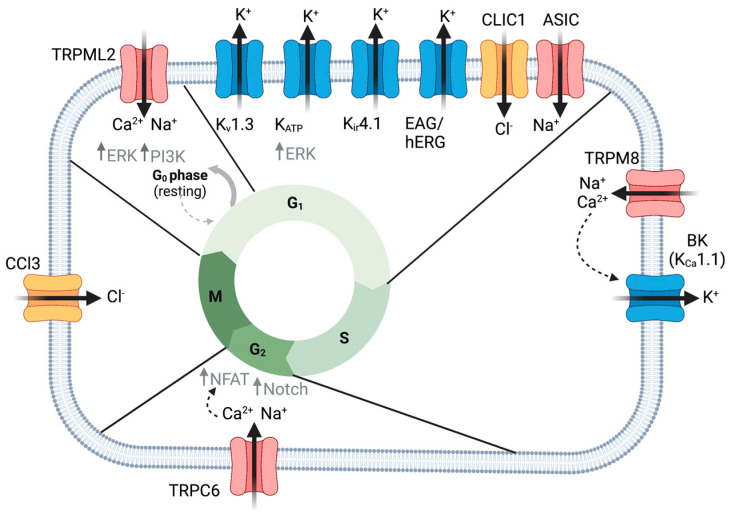
Ion channels regulate glioma cell proliferation. TRPML2 mediates exit from quiescence via increased ERK and PI3K growth signaling. Several K^+^ channels, as well as CLIC1 and ASIC channels, are important in the G1 to S phase transition of the cell cycle. KATP promotes ERK growth signaling. TRPM8 increased intracellular Ca^2+^ important for activating BK channels driving S to G2 cell cycle transition. TRPC6-mediated Ca^2+^ enhances Notch signaling and NFAT translocation to the nucleus, promoting G2 to M phase transition. CCL3-mediated premitotic condensation is important for proliferation. Created with BioRender.com.

Cellular proliferation is required to build and maintain organisms throughout life. It is a tightly regulated process involving cell growth followed by division into two cells. Cell cycle progression is tightly regulated as its dysfunction, a hallmark of cancer, can lead to uncontrolled growth and tumor formation [5,43]. It is becoming increasingly clear that ion channels are important regulators of proliferation and cell cycle progression (Figure 1, Table 2). Actively proliferating cells tend to be more depolarized compared to differentiated tissue [44], and depolarization itself has been found to drive proliferation. Studies from the 1970s onward have found that the membrane potential fluctuates during the cell cycle [44,45]. These studies performed in cell culture of Chinese hamster ovaries (Cho cells) and the breast cancer cell line MCF found a tendency for cells to hyperpolarize during the G1/S transition, depolarize toward G0/G1 and again hyperpolarize toward the G2/M transitions [44,46,47]. Much of what we know about ion channel dynamics is derived from excitable cells, and while ion channels are implicated in glioma proliferation, we know considerably less about their role. Defining the role of specific ion channels and the electrophysiological dynamics underlying glioma cell behavior is necessary to understand their functional significance. Open questions include: (1) which ion channels are involved in the regulation of cellular membrane potential dynamics? (2) How do bioelectric changes influence intracellular signaling pathways? (3) Are there bioelectric signatures unique to oncogenic proliferation?

### 2.1. K^+^ Channels

Potassium channels (K^+^) are a diverse class of ion channels that regulate various functions across cell types. One important role regulated by K^+^ channels includes establishing the resting membrane potential of cells. A number of K^+^ channels are upregulated in human glioma biopsies [11,32,61,65,129,130,131,132,133,134,135,136] as well as glioma cell lines [27,32,33,58,65,66,132,133,134,137,138]. The expression of these K^+^ channels, in some cases, correlates with more aggressive tumors and worse survival rates, whereas others are linked to low-grade gliomas (Table 1). A wide variety of K^+^ channels are enriched in gliomas, including: voltage-gated K^+^ channels (Kv), Ca^2+^-activated K^+^ channels (Kca), inward rectifying K^+^ channels (Kir), and ATP-sensitive K^+^ channels (KATP). These channels have different gating properties and downstream targets, suggesting that they may mediate a wide range of cellular behaviors (Table 2).

Abnormal expression of K^+^ channels is associated with the proliferation of gliomas (Table 1). To determine if they directly regulate proliferation, researchers have manipulated K^+^ channel expression and used K^+^ channel-specific drugs to influence activity. Numerous studies implicate K^+^ channels in G1/S cell cycle progression. Ru et al. examined a suite of K^+^ channel blockers on proliferation in U87MG cells [138]. 4-Aminopyridine (4-AP) is selective for Kv1 voltage-gated channels including Shaker K^+^ Channels (*KCNA3*, Kv1.3). Tetraethylammonium (TEA) is a general K^+^ channel blocker and glibenclamide, a widely used antidiabetic drug, selectively blocks KATP channels. All of these K^+^ channel antagonists were sufficient to reduce proliferation and shift cells to G0/G1 and away from the S phase [138,139]. This is consistent with the roles of K^+^ channels in facilitating the progression of the G1/S checkpoint. We will further discuss studies examining K^+^ channel subtypes in the following sections.

#### 2.1.1. hERG and EAG

Voltage-gated K^+^ channels, human ether-a-go-go-related channel (hERG, also known as Kv11.1 and encoded by the gene *KCNH2*), and hEAG (human ether a-go-go, Kv10.1, *KCNH1*) are expressed in gliomas where they have been shown to control proliferation and apoptosis [140]. EAG expression was highly enriched in brain metastases of 75% of patients with glioblastoma as opposed to the primary tumor [9]. Lower hEAG expression in either primary or glioma metastases was correlated with increased survival [9]. Pointer and colleagues examined hERG expression in 115 glioblastoma patient-derived cells (GPDC) and found that hERG expression was correlated with worse patient survival outcomes [10]. hERG expression was correlated with increased proliferation (identified by Ki-67 expression) in both sphere formation assays and mouse xenograft models. hERG inhibitor, E-4031, was sufficient to decrease proliferation [10]. Notably, blocking hERG with one or more inhibitors increased patient survivorship (Table 2) only in patients with increased hERG expression [10]. Consistent with this, hERG blockers doxazosin, a blood-pressure-lowering medication, and letrozole, an aromatase inhibitor used in certain types of breast cancer, were found to reduce proliferation in multiple glioma cell lines (LNT-229, U87MG, and U373) [58,59]. Staudacher and colleagues similarly found that shRNA knockdown of hERG led to cell cycle arrest and apoptosis in human LNT-229 and U87MG glioblastoma cells. Cell cycle analysis indicated that doxazosin-mediated blocking of hERG led to cell cycle arrest in the G0/G1 phase (Figure 1) [58]. Together, these data suggest that hERG channels may be a biomarker for aggressive gliomas and a potential target to improve survival in glioblastoma patients.

#### 2.1.2. KATP Channels

Compared to non-tumor control cells, KATP expression was enriched in both glioma cell lines and patient glioma tissue samples [60]. Applying KATP antagonist and antidiabetic drug tolbutamide, Huang and colleagues found that the proliferation of glioma cells was decreased, and the cell cycle was arrested at G1/G0 (Figure 1) [60]. KATP activation with agonist diazoxide reduced the number of cells in G1/G0 and increased the proportion in the S phase. In addition, activating KATP in a mouse glioma explant model further enhanced tumor growth. Huang et al. determined that KATP-enhanced glioma diazoxide reduced the number of cells in G1/G0 and increased the proportion in the S phase. In addition, activating KATP in a mouse glioma explant model further enhanced tumor growth. Huang et al. determined that KATP enhanced glioma growth by activating ERK proliferation pathways [60]. Overall, these experiments indicated that KATP channels regulated cell cycle progression and glioma growth [60].

#### 2.1.3. Ca^2+^-Activated K^+^ Channels

Ca^2+^-activated K^+^ channel BK (big conductance) channel (Kca1.1 encoded by *KCNMA1*) expression level was positively correlated with the degree of glioma malignancy [11]. TEA blockage of BK channels inhibited proliferation and led to apoptosis of C6 glioma cells and 9L glioma cells [61]. These cells were arrested at G1/G0 (Figure 1) and displayed increased levels of reactive oxygen species (ROS) accompanied by an upregulation of p53 and CDK inhibitor p21 [61]. A later study by Abdullaev et al. identified functional expression of BK and Intermediate conductance (IK, Kca3.1) and Small conductance (SK) Ca^2+^-activated K^+^ channels in glioblastoma cell lines (U251 and U87MG) as well as a surgical sample of glioblastoma [141]. While they also found that inhibitors of BK (paxilline and penitrem A) and IK1 channels (clotrimazole and TRAM-34) were sufficient to decrease proliferation, there was no effect on SK channels when blocked with inhibitor UCL1848. Moreover, they noted that the concentrations necessary for blocking proliferation were higher than those necessary for blocking these ion channels. When they used lower concentrations or knocked down these channels with siRNA, proliferation was unaffected. The authors concluded that high concentrations of K^+^ channel blockers inhibit proliferation through off-target effects. Thus, the role of BK in proliferation remains unresolved. However, this group of channels is highly implicated in other aspects of glioblastoma, including infiltration and cellular migration, which will be discussed in Section 3.

Interestingly, Stegen et al. found that IK channels did not influence proliferation in T98G glioblastoma cells. However, upon cellular irradiation, IK channel activity was important for cell cycle progression. Specifically, blockage with inhibitor TRAM34 or RNAi knockdown impaired cell cycle control and induced G2/M arrest [27]. Thus, radiation was found to induced IK expression. Manipulation of IK with TRAM34 was also found to be important for regulating baseline Ca^2+^ concentrations and Ca^2+^ oscillations. One possible explanation for the influence of IK on proliferation could be through Ca^2+^ signals. Ca^2+^ dynamics have been correlated with cell cycle progression and irradiated disruption of Ca^2+^ effector CamKII (Calmodulin Kinase II) has been found to induce G2/M arrest via inactivation of phosphatase cdc25B, an inactivator of cdc2 in its phosphorylated inactive form [27]. G2/M phase is an important cell cycle check point for DNA damage and subsequent repair, as damaged chromosomes can lead to mitotic catastrophe and cell death [67,142].

#### 2.1.4. Kir4.1

Kir channels are expressed in gliomas, but they do not traffic to the cell membrane [31,143]. This indicates that gliomas may alter Kir localization to promote their growth. Indeed, gliomas tend to be depolarized. As mentioned above, a depolarized state is common among proliferating tissues where a decreased membrane potential supports an alkaline intracellular pH and increased Ca^2+^ signaling, processes that are both conducive to cell cycle progression [144,145]. Higashimori and colleagues found that overexpression of Kir4.1 in D54-MG glioma cell lines, hyperpolarized glioma cells and shifted them from G2/M to a non-proliferative G1/G0 state (Figure 1) [48]. Thus, gliomas may downregulate Kir4.1 to maintain a depolarized state that is permissive for proliferation.

### 2.2. TRP Channels

The Transient receptor potential channels (TRP channels) are nonselective cation channels composed of six transmembrane segments that facilitate diverse roles in sensing and responding to external stimuli [91]. There are currently nine proposed families split into two groups. Group 1 includes TRPC (canonical), TRPV (vanilloid), TRPVL (vanilloid-like), TRPM (melastatin), TRPS (soromelastatin), TRPN (no mechanoreceptor potential C), and TRPA (ankyrin). Group 2 includes TRPP (polycystic) and TRPML (mucolipin). Alptekin et al. found by qRT-PCR broad expression of TRP channels, including TRPC1, TRPC6, TRPM2, TRPM3, TRPM7, TRPM8, TRPV1, and TRPV2 that were significantly higher in glioblastoma patient samples (Table 1) [146]. Moreover, these authors identified a positive correlation between high expression with patient survival. However, subsequent studies identified a subset of TRP channels negatively correlated with tumorgenicity [12,13,147]. There is a growing consensus that TRPV1, TRPV2, TRPM2, TRPM3, and TRML1 exert anti-tumorigenic properties in glioma, while TRPC1, TRPC6, TRPM7, TRPM8, and TRPML2 promote glioma growth and proliferation.

#### 2.2.1. TRPV1 and TRPV2

Amanti et al. observed that TRPV1 expression was inversely correlated with glioma grading based on studies in NHA (normal human astrocytes), U87MG, and U373 glioma cell lines [12]. Moreover, in patient samples, the *TRPV1* gene and protein expression were inversely correlated with glioma grading, and a near complete loss of TRPV1 expression was found in grade IV glioblastoma [12]. TRPV1 may be downregulated by gliomas resulting in protection against apoptosis. This is suggested by studies that found treatment with TRPV1 agonist capsaicin, increased intracellular Ca^2+^ concentrations, which in turn may induce apoptosis through activation of p38 MAPK [12]. Consistent with this hypothesis, TRPV1 is downregulated in high-grade astrocytoma cells [148]. Nabissi and colleagues identified the downregulation of TRPV2 in high-grade gliomas as well as in U87MG cells and in MZC, FCL, and FSL primary glioma cells [13]. Reducing the expression of TRPV2 with siRNA knockdown enhanced the proliferation of U87MG glioma cells indicating TRPV2 negatively controls glioma proliferation [13]. On a molecular level, knockdown led to the modulation of cell cycle- and apoptosis-related genes. Specifically, increased expression of cyclin E1, cyclin-dependent kinase 2, E2F1 transcription factor 1, Raf-1, and anti-apoptotic bcl-Xl genes was observed, while the expression of pro-apoptotic genes Fas and pro-caspase-8 was reduced [13].

#### 2.2.2. TRPM2 and TRPM3

Both TRPM2 and TRPM3 have demonstrated protective roles in glioma; however, TRPM2 has been more thoroughly studied [91]. TRPM2 is activated by hydrogen peroxide and is considered a sensor of oxidative stress [149,150]. Transfection of TRPM2 in A172 human GBM cells or activation of TRPM2 in DBTRG GBM ( cells both led to increased intracellular Ca^2+^ concentrations and cell death [92,93].

#### 2.2.3. TRPML1

TRPMLs are expressed in lysosomes and are involved in autophagy regulation. Of the three evolutionarily related TRPML channels (TRPML1-3), only TRPML1 has been characterized in the context of endolysosomal Ca^2+^ signaling and cancer behavior [151]. TRPML1 may play a protective role against glioma progression. This is suggested by the work of Morelli and colleagues, who showed that activation of TRPML1 by its agonists MK6-83 induced caspase-3-dependent apoptosis in T98G and U251 glioma cell lines. Furthermore, knockdown or pharmacological inhibition of T98 and U251 both led to reduced intracellular Ca^2+^ concentrations and restored glioma cell line viability [101]. Inducing ROS within glioma cells also triggered autophagic cell death that may be mediated through TRML1 lysosomal Ca^2+^ release [101]. Thus, stimulation of TRPML1 could lead to cell death through induction of apoptosis via increased Ca^2+^ or through its actions as a ROS sensor triggering autophagy [101].

#### 2.2.4. TRPC1 and TRPC6

Work by Bomben and colleagues implicated TRPC channels as regulators of proliferation in GBMD54MG glioma cell lines [88]. They discovered that chronic application of TRPC inhibitor SKF96365 led to cell cycle arrest at the G2/M stage as well as abnormal cell division [88]. GBMD54MG cells displayed defective cytokinesis resulting in large multinucleated cells [88]. TRPC1 seems to mediate the Ca^2+^ dynamics required for cytokinesis. shRNA knockdown of TRPC1 in GBM D54MG led to similar cytokinesis effects as a pharmacological blockage. This indicates that TRPC1 channel activity mediates its role in cell division [86]. Acute application of SKF96365 revealed a role in TRPC control of resting membrane potential. SKF96365 application induced a transient hyperpolarization, followed by a sustained depolarization [88]. Hypoxia is linked to aggressive glioblastoma progression. GBM patient samples displayed increased TRPC6 and Notch expression. Chigurupati and colleagues found that hypoxia induced both Notch and TRPC6 expression [14]. TRPC6 expression required Notch as blocking Notch activation prevented TRPC6 expression. Mechanistically, TRPC6 expression led to increased steady-state Ca^2+^, which led to increased NFAT expression in MG373 glioma cell lines [14]. NFAT is a transcription factor known to be essential for glioma proliferation [152]. Blocking TRPC6 or NFAT under hypoxic conditions reduced glioma cell line proliferation [14]. Another study found that TRPC6-dependent Ca^2+^ was required for G2/M cell cycle progression (Figure 1) [89].

#### 2.2.5. TRPM7 and TRPM8

TRPM7 and TRPM8 have both been linked to glioma proliferation. Knockdown of TRPM7 expression upregulated tumor suppressor miR-28-5p across multiple glioma cell lines [94]. Of note, the microRNA miR-28-5p has previously been shown to influence tumor growth by suppressing AKT, ERK, and IGF-1 signaling pathways [153,154]. Further, Liu and colleagues determined that this regulation required TRPM7 channel activity. miR-28-5p suppressed glioma cell proliferation by targeting Rap1b [155]. TRPM7 was also implicated in the regulation of additional growth pathways, including Notch, STAT3/ALDH1 in glioma cell lines [95,96]. TRPM8 inhibition or knockdown was sufficient to impair the cell cycle resulting in apoptotic cell death that may be mediated by MAPK signaling, ERK, and BCL2 in human glioma cells [97,98]. Ca^2+^ from TRPM8 is thought to regulate the cell cycle by driving S phase progression and mitosis (Figure 1) [97]. TRPM8 may further regulate membrane potential and cell cycle progression by mediating the expression of Kir4.1 [99].

#### 2.2.6. TRPML2

Limited studies provide evidence for the role of TRPML2 in the regulation of glioma cell survival and proliferation. Suppression of its expression impaired cell cycle progression, reduced proliferation, and decreased glioma cell viability [103]. Silencing TRMPL2 resulted in the inactivation of canonical growth signaling pathways: PI3K/AKT and ERK1/2. Being involved in the regulation of proliferation and apoptosis, these pathways may mediate glioma cell growth related to TRPML2 [102].

### 2.3. Chloride Channels

Cl^−^ channels are important for membrane potential stabilization, regulation of Ca^2+^ signaling, and volume control in normal tissues as well as glioblastoma cells [156]. Cl^−^ channel enrichment in glioblastoma cell lines and human glioblastomas indicated their importance for the pathophysiology of tumor progression. Moreover, experiments implicated Cl^−^ channels in volume regulation important for glioma cell proliferation, viability, migration, and infiltration (Table 2) [156].

#### 2.3.1. CIC

Chloride channels (ClC) are an evolutionarily conserved family of voltage-gated Cl^−^ channels that are structurally unrelated to other classes of voltage-gated ion channels [157]. There are nine of these channels broadly expressed in mammals that are localized to the plasma membrane or within intracellular membranes. ClC3 was found to mediate premitotic condensation of malignant glioblastoma cell lines (D54-MG and U251-MG) [81,82,158]. Premitotic condensation is a cytoplasmic reduction in cell size that is linked to chromatin condensation. Perturbing this condensation delayed cell division [81,82,158]. During the M phase, ClC3 was found to localize to the plasma membrane and mitotic spindle [82]. In addition, an increased Cl^−^ current was found during the M phase using patch clamp electrophysiology, and this was abolished by shRNA knockdown of ClC3 [82].

#### 2.3.2. VRAC

VRAC (volume-regulated anion channel) mediates the swelling-activated chloride current (IClswell) and is highly expressed in GBM cells [15,16,17]. Glioma cells are often found in a hypoxic environment. Sforna et al. found that VRAC was important for proliferation and cell survival in hypoxia [85]. Their studies showed that the regulatory volume decrease following a hypoxia-induced increase in cell volume is mediated by VRAC. Blocking VRAC with antagonist DCPIB (4-[(2-Butyl-6,7-dichloro-2-cyclopentyl-2,3-dihydro-1-oxo-1H-inden-5-yl)oxy]butanoic acid) prevented this volume decrease and led to necrotic cell death [85]. Cell volume changes are also important for proliferation. Wong and colleagues observed slowed proliferation of U251 GBM cells related to reduced PI3K/Akt/mTor signaling in the presence of the VRAC inhibitor DCPIB [159]. In contrast, Liu and colleagues found VRAC to be dispensable for proliferation in glioblastoma cells (GBM cell lines U251 and U87MG) [160]. The apparent discrepancy between these studies related to the effects of VRAC expression on glioma proliferation needs further clarification.

#### 2.3.3. TMEM16A

TMEM16A (transmembrane protein with unknown function 16), also known as ANO1 (anactomin-1), is a Ca^2+^-activated Cl^−^ channel. The mRNA expression levels of TMEM16A increased with higher glioma grades, with particularly high levels observed in grade III and IV gliomas [18]. A study by Kim et al. demonstrated a negative correlation between TMEM16A expression and patient survival for the gliomas with the highest TMEM16A expression levels (top 10%) (Table 1) [19]. TMEM16A expression was also high in multiple glioma cell lines (U87MG, U118, U251, and SHG44) [18]. siRNA knockdown of TMEM16A decreased the proliferation of U87MG glioma cell lines. Using a luciferase assay, Liu and colleagues provided evidence for TMEM16A-mediated modulation of glioma proliferation by showing that TMEM16A promotes NF-κB-mediated gene transcription, leading to increased levels of cell cycle regulators cyclin D1, cyclin E, and c-myc [18]. A study by Kim et al. found that TMEM16A acted to stabilize EGFR signaling in glioma stem cells to support stemness and tumor progression. Knockdown of TMEM16A reduced self-renewal of glioma stem cells and stem cell factors EGFRvIII, Notch, Nestin, and Sox2. Further, the reduction of TMEM16A led to increased survival of an intracranial glioma mouse model [19], highlighting TMEM16A as a potential glioma biomarker and target for therapeutic intervention [161].

#### 2.3.4. CLIC

CLIC (Cl^−^ intracellular channel protein) consists of six family members CLIC1-6. CLIC1 has been linked to glioma proliferation. CLIC1 is overexpressed in GBM compared with normal tissues, and its expression was associated with a worse prognosis (Table 1) [20]. CLIC1 was identified as a circulating protein secreted in extracellular vesicles (EVs). Treatment of GBM cells with EVs derived from CLIC1-overexpressing GBM cells strongly induced proliferation both in vitro and in vivo [162]. Investigation of the basic functions of GBM CSCs reveals a constitutive state of oxidative stress and cytoplasmic alkalinization compared with MSCs (mesenchymal stem cells). Both intracellular oxidation and cytoplasmic pH changes have been reported to affect CLIC1 membrane functional expression [80]. Blocking CLIC1 led to a reduction in ROS accumulation and increased intracellular acidity, which the authors predict prevented cell cycle progression from the G1 to S phase (Figure 1) [80].

### 2.4. NHE

NHE (Na^+^/H^+^ exchanger) are major regulators of intracellular pH encoded by nine human family members. NHE5 and NHE9 are both linked to glioma proliferation. C6 glioma cells were found to express high levels of NHE5 [21]. Highly proliferating cancer cells tend to be more alkaline and hypoxic, which supports glycolytic metabolism (see Section 4, Glioma Metabolism). Glycolysis generates high levels of lactate, which increase H^+^. Increased H^+^ would activate NHE5 to pump out H^+^ and maintain a more alkaline environment. Glioma xenografts originating from *NHE5*-knockdown cells exhibited significantly slower growth than those from *NHE1*-knockdown cells and control cells [21]. This implied a specific role for NHE5 in glioma growth potential. Kondapalli et al. identified microRNA 135a (miR-135a) as targeting NHE9 and reducing its expression [116]. Expression of miR-135a was reduced in U87MG glioma cells compared to non-tumor cells [116]. Conversely, increased expression of miR-135a in U87MG glioma cells or direct knockdown of *NHE9* via siRNA or drug blockage led to reduced proliferation of glioma cells. High NHE9 expression was associated with attenuated receptor turnover of EGFR [117]. EGFR is commonly upregulated in gliomas and leads to uncontrolled proliferation. Kondapalli and colleagues suggested that NHE9 may contribute to aberrant EGFR signaling [117].

### 2.5. ASIC

ASIC (acid-sensing ion channel) sense and are activated by acidic extracellular pH, leading to cation permeability (primarily Na^+^, in some cases Ca^2+^). Expression of ASIC1a and ASIC3 has been detected in primary GBM stem cell lines [163]. ASIC1 expression levels in GBM tumor tissues were lower than expression in normal brain tissue. Glioma patients with high ASIC1 expression had longer survival than those with low ASIC1 expression, pointing to ASIC1 as a potential prognostic biomarker for survival in GBM (Table 1) [22]. Functional studies by King and colleagues found that the knockdown of ASIC1a suppressed the growth and proliferation of glioma cells (A172 as well as U87MG) through G1/S arrest and apoptosis induction (Figure 1). Mechanistically, ASIC1a negatively modulated glioma stemness via inhibition of the Notch signaling pathway and GSC markers CD133 and aldehyde dehydrogenase 1 [22]. Ross and colleagues identified a role for ASIC1 in glioma cell volume regulation in response to a hyperosmotic solution [106]. Glioma cells first swell, then shrink in response to hyperosmotic solutions, but this was blocked when cells were administered ASIC1-specific toxin blocker psalmotoxin1 [106]. This may indicate a role for ASIC1-mediated volume regulation during glioma cell cycle progression.

### 2.6. PIEZO

Cells are exposed to various mechanical forces depending on their environment. For cancer cells, including gliomas, mechanical aspects play roles in shaping the tumor microenvironment (TME). Solid tumors are recognized for their altered mechanical properties compared to non-tumor tissue, with emerging research finding that tumors are composed of a heterogenous mix of cells with varying rigidity [164]. Indeed, there is a growing body of research advancing our understanding of how increased tissue stiffness contributes to malignant progression [165]. The degree of tissue stiffness has been found to facilitate certain types of cancer cell behavior. For instance, it was found that cells became softer by downregulating keratin following epithelial–mesenchymal transformation [166,167]. This change to a “squishier” consistency may facilitate cell migration through dense tissue environments [168]. Tumor cells can become stiffer by stimulating fibrosis through the enhancement of the extracellular matrix [169]. Fuhs et al. recently demonstrated that tissue stiffness could also result from the accumulation of migrating cells getting stuck [164]. This phenomenon is termed nuclear jamming due to the rigid nature of the nucleus restricting cellular movement [170]. The accumulation of jammed cells provides mechanical tumor support, whereas the unjammed cells continue to migrate [164].

Regulation of altered tumor tissue mechanics require sensing of mechanical forces in the environment. PIEZO is a mechanotransduction channel with permeability to Na^+^, K^+^, and Ca^2+^. PIEZO1 is overexpressed in aggressive human gliomas, and its expression inversely correlates with patient survival [23,24]. Chen and colleagues used both *Drosophila* and mouse models to identify a role for mechanosensitive channel PIEZO in glioma progression [23]. dPiezo increased tissue stiffness in *Drosophila* glioma models but not in normal tissues. Moreover, they found that this increased stiffness led to increased activation of dPiezo, resulting in Na^+^, Ca^2+^, and K^+^ flux. dPiezo localized to focal adhesions where it genetically interacted with integrin signaling to reinforce tissue stiffening and promote glioma aggression. This phenomenon was conserved to mammals as the knockout of *PIEZO1* suppressed the growth of glioma stem cells, inhibited tumor development, and extended the survival of a murine xenograft model [23].

### 2.7. Voltage-Gated Na^+^ Channels

Actively proliferating cells, including cancer cells, have been found to exhibit a more depolarized state [44,171]. Voltage-gated sodium channels (VGSC) are major drivers of neuronal depolarization, but their influence on brain tumors is largely undefined. Recent work examined the role of VGSC in *Drosophila* and found that RNAi knockdown of *para*, the sole VGSC in the fly, reduced the tumor size of three distinct brain tumor models [172]. Para was also found to regulate the proliferation of neural progenitors that was dependent on its ion channel activity. Going forward, it will be interesting to determine whether membrane depolarization or Na^+^ concentrations are general regulators of proliferation or if they specifically promote oncogenic processes.

### 2.8. AMPAR

Recent findings discovered that neurons made synaptic connections with glioma cells and that glutamate transmission mediated by AMPARs through these synapses promoted proliferation [127]. Blocking neural activity with voltage-gated sodium channel toxin TTX (tetrodotoxin) or glutamate release via cystine/glutamate exchange (inhibited by sulfasalazine (SAS)) in co-cultures reduced proliferation, but this was not seen in glioma monocultures. This work described a novel mechanism of glioma growth regulation through neural-glial glutamatergic synapses [42,127].

## 3. Infiltration and Cellular Migration

**Figure 2:**
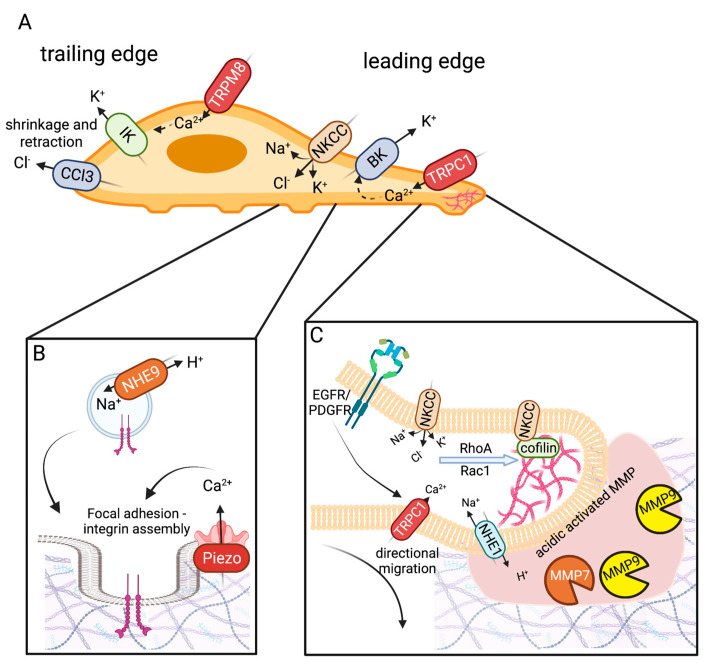
Ion channels mediating glioma infiltration and mesenchymal migration. (**A**) Migrating cells have a leading and trailing edge. The trailing edge shrinks and retracts, mediated by IK and CLC3 ion channels. The leading-edge swells (mediated by BK channels) establish focal adhesions (PIEZO and NHE9) for traction and utilizes F-actin polymerization (NKCC) to establish new cellular territory. (**B**) Focal adhesions assembly involves localization and clustering of β-integrin to the plasma membrane. NHE9 acts as a H^+^ leak establishing a pH state important for endosomal protein trafficking of β-integrin. Focal adhesion formation is promoted by PIEZO1-mediated mechanosensation. This contributes to glioma stiffening that creates a feedback loop promoting glioma migration and metastasis. (**C**) The leading edge of migrating cells with invadopodium express NHE1, which acidifies the extracellular matrix (ECM)-activating matrix metalloproteinases MMP-7 and MMP-9. MMPs break down the ECM to allow migrating glioma cells to invade new territory. NKCC restores K^+^ and Cl^−^ levels following cell shrinkage. It also activates cytoskeletal remodeling of the leading edge by promoting the actions and localization of RhoA, Rac1, and Cofilin. NKCC-mediated actin polymerization is important for lamellipodium extension and directional migration in response to chemotaxis. Growth factors EGF and PGDF act as chemoattractants via activation of EGFR/PDGR signaling. Their downstream effects promote localization of TRPC1 (and likely other TRPC channels) which is thought to influence directional migration. Created with BioRender.com.

Glioblastoma (GBM), the most aggressive form of glioma, is a common primary tumor that exhibits poor patient prognosis due to its invasive nature and resistance to treatment. While glioblastomas almost never metastasize out of the brain, extensive infiltration of GBM cells into the brain parenchyma is a hallmark of the disease [173]. The inability to distinguish clear boundaries between tumor tissue and healthy tissue contributes to the high mortality rate of GBM patients [174]. Common GBM treatments involve surgery, radiation, and temozolide treatment; however, within 2 years of treatment, nearly all patients experience regrowth [174]. Thus, there is an unmet need for new treatments to limit the invasiveness and improve patient outcomes of GBM. Ion channels are emerging as major regulators of GBM cell invasion and migration. Achieving a better understanding of their physiological role in these processes may lead to novel treatments desperately needed for this devastating disease. Parenchyma infiltration involves migration from the site of the primary tumor, followed by invasion and infiltration into new brain territory (also discussed in Section 5, Tumor Microenvironment). We will review the molecular contributions of ion channels to these processes (Table 2, Figure 2).

GBM cells migrate along major structural components of the brain, blood vessels and white mater tracts [62,174]. Gliomas are composed of heterogeneous cell types with varied expressions. There are two primary modes of cellular migration: mesenchymal and amoeboid [175]. Previously, most studies examining glioma migration utilized 2D cell cultures. However, the native environment infiltrated by glioma cells is 3D and composed of dense, complex cell types. Recent work has transitioned into using 3D glioma cultures [164,176] or has examined glioma explants in rats or mice using 2-photon microscopy, which permits greater depth of imaging within an intact brain [176] to achieve a more accurate view of glioma migration. One such study, by Koh et al., found that glioma cells in 3D culture exhibited primarily two morphologies: elongated (58.7%) and rounded (41.3%) [177]. These cellular morphologies were correlated to specific molecular signatures and migration modes. Elongated cells moved via mesenchymal mechanisms involving rearrangement of the cytoskeleton with leading and trailing edges that propelled movement. Rounded cells exhibited amoeboid movement that was less dependent on ECM remodeling [177]. Intriguingly, glioma cells were able to adjust their mode of migration in response to an altered cellular environment. This work clearly indicates that glioma cells utilize multiple modes of invasion and infiltration. Much of the discussion here focuses on mesenchymal modes of migration, as it represents the best-described mechanism linked to ion channels. However, it is essential for future ion channel-related studies to investigate amoeboid modes of movement, which include blebbing and pseudopodia, as these modes were observed in nearly half of the cell types in the 3D glioma culture [177]. Ion channels likely play roles in these processes that are currently undefined and may represent a new avenue for therapeutic intervention. PIEZO, a mechanosensitive channel reviewed above, has been implicated in amoeboid-style movement involving blebbing in other cell types [178,179] and is briefly discussed from this perspective below.

Mesenchymal cellular migration requires cytoskeletal rearrangement involving cell protrusions and polarization of cells with a leading and trailing end. Cellular protrusions include: invadopodia, lamellipodia, and filopodia [180]. Invadopodia are F-actin-rich protrusions that extend into the extracellular matrix (ECM). These protrusions use enzymatic activity to degrade the extracellular matrix, making room for migrating cells [180]. Lamellipodia are flat, broad membranous projections located at the leading edge of a cell. These protrusions attach through cell surface proteins to cells within the ECM and use actin polymerization to generate force to propel the cell forward [181]. Filopodia are thin finger-like projections composed of parallel actin bundles. They function similarly to lamellipodia but are more exploratory and play roles in the directional movement of migrating cells [180,181]. Mesenchymal cellular movement progresses as the leading edge protrudes and swells while the trailing end shrinks and retracts [62]. Live imaging research has found that migrating glioma cells undergo dramatic changes in shape and size, reducing their volume by up to 33% in order to navigate through tight spaces within the brain parenchyma [182]. This substantial shape change is mediated by water flow through aquaporins AQP1 and AQP4 (discussed in Section 5, Tumor Microenvironment) and is guided by ion flux of primarily K^+^ and Cl^−^ channels [182]. KCa1.1 (BK), NKCC1 (Na^+^/K^+^/Cl^−^ transporter 1), and TRPC1 are enriched at leading edges where they facilitate efflux of K^+^ in response to Ca^2+^ activation and Na^+^, Cl^−^ influx along with K^+^ efflux, respectively [174]. The synergistic actions of these channels are thought to orchestrate volume changes to promote tumor cell invasion into tight areas and to propel cellular migration.

### 3.1. Ion Channels and Exchangers Enriched in Glioma Cell Leading Edge

#### 3.1.1. NKCC1

NKCC1 expression was increased in high-grade glioma compared to lower-grade glioma (grade II) [25,26]. NKCC1 expression in primary GBM cells localized primarily to the leading edge (Figure 2) [26]. Glioma cells exhibit a large Cl^−^ gradient with intracellular Cl^−^ concentrations about 10x higher (~100 mM) than that of neurons [182]. The Cl^−^ gradient is thought to be one of the major driving forces underlying glioma size and shape changes during infiltration and migration [182]. The Cl^−^ gradient is generated by the high expression of NKCC1 in glioma cells. Similar to most cells, K^+^ is highly concentrated within the cell. Thus, when K^+^ or Cl^−^ channels open, there is a large outward driving force for these ions which drives water molecules to flow down their osmotic gradient, effectively reducing cell volume [182]. Subsequent NKCC1 activation restores cellular volume. The ability to alter cell volume is thought to aid migrating cells in navigating tight spaces. Work by Garzon et al. found that NKCC modulated glioma invasion through focal adhesion dynamics and cellular contraction [25]. Lamellipodia and filopodia are actin-rich protrusions that drive cellular migration [174]. Knockdown of NKCC1 led to larger focal adhesions with lower cell traction forces [25]. NKCC1 was also found to regulate migration through the modulation of multiple downstream targets that influence F-actin [26]. NKCC1 mediated localization of Cofilin-1 [26]. Cofilin-1 cuts actin filaments to facilitate new actin polymerization. These processes are important for determining the direction and promotion of lamellipodium extension. Cofilin-1 acts together with Rho and Rac GTPases to regulate cytoskeletal rearrangement required for cellular migration. NKCC was found to act as a scaffold to Cofilin-1, targeting it to the leading edge of the migrating GBM cell. Knockdown of NKCC1 led to a reduction in both Cofilin-1 and active RhoA and Rac1, effectively reducing formation of filamentous actin, cellular protrusion, and migration (Figure 2A) [26].

#### 3.1.2. BK Channels

BK channels were found to be highly expressed in GBM-stem-like cells, where they facilitated high levels of migration [65]. BK channels are localized to leading-edge filopodia [62]. Activation of BK increased K^+^ efflux, which promoted entry of Cl^−^, Na^+^, and H_2_O [63,64,65,66]. Swelling of the leading edge helped promote GBM cell migration (Figure 2A). Interestingly, hypoxia, a frequent characteristic of the tumor microenvironment, was found to increase the migratory ability of U87MG cells in a BK-dependent manner [133]. Together, this work suggests that BK channels may have an important role in glioma cell migration.

#### 3.1.3. TRPC

TRPC1 was implicated in glioma cell migration in response to growth factors EGF (epidermal growth factor) and PDGF (platelet-derived growth factor) [87,183]. Stimulation of EGF resulted in TRPC1 localization to the leading edge of D54-MG cells, which was dependent on the integrity of lipid rafts [87]. Interestingly, generalized TRPC blocking resulted in a complete loss of chemotaxis. However, specific knockdown of TRPC1 compromised directional migration with preserved cellular motility [87]. Overall, current studies support an EGF-dependent role of TRPC1 on glioma on migration via chemotactic attraction toward EGF, while basal glioma cellular motility does not seem to be TRCP1 dependent. Furthermore, other TRPC channels are likely contributing to the EGF-dependent chemotaxis (Figure 2B). Localization of TRPC1 to the leading edge of migrating GBM cells was found in response to PDGF stimulation of U251 GBM cells [183]. TRPC6 has also been implicated in GBM invasiveness under hypoxic conditions [14,89]. However, whether these findings indicate more direct roles of TRPC channels in glioma cell motility and migration, in addition to mediating growth factor-mediated chemotaxis, remains to be investigated further.

### 3.2. Ion Channels Enriched in Trailing Edge

#### 3.2.1. KCa3.1 (IK)

While normal human astrocytes displayed low expression of Ca^2+^-activated K^+^ channel KCa3.1 (IK), GBM cells functionally expressed high levels of IK during malignant transformation [27]. The expression of IK channels in GBM was negatively associated with patient survival (Table 1). Patch clamp recordings have revealed the expression of IK channels at the trailing edge of T98G, U87MG, and U251 cells and where these channels were thought to mediate migration and invasion [27,67]. Catacuzzeno and colleagues identified IK expression at the invadopodium of D54 human glioma cells [16,17]. Blocking IK with specific inhibitor TRAM-34 prevented Ca^2+^ oscillations and chemotaxis of D54 GBM cells [16], as well as bradykinin-induced chemotaxis of U87MG cells [68,69,70]. The same blockage was found to prevent GBM invasion of U251 cells and primary glioblastoma neurosphere cultures into brain slices [71,72].

#### 3.2.2. CLC-3

GBM cells exhibit increased expression of voltage-gated Cl^−^ channel 3 (CLC3) compared to normal brain tissue. Blocking the channel with non-specific Cl^−^ channel blocker or specific RNAi knockdown both led to reduced glioma cell migration [83]. Additional support for CLC3 channel activity mediated cellular motility came from research investigating CLC3 regulator CaMKII [69]. Kinase CaMKII phosphorylation of CLC3 enhanced Cl^−^ currents and shRNA of CLC3 or inhibition of CaMKII both reduced glioma cell invasion [69]. Along with IK channels, CLC3 was found to localize to the trailing edge of glioma cells, where it was thought to regulate cellular shrinkage and retraction through H_2_O efflux (Figure 2A,C) [70]. Further defining the mechanistic context for CLC3 in glioma cells, Wang and colleagues demonstrated that shRNA knockdown of CLC3 decreased volume-related Cl^−^ currents and nuclear translocation of NF-kB [84]. Reduced NF-kB led to a decrease in MMP-3 and MMP-9 expression, proteinases involved in remodeling of the ECM essential for invasion [84,174].

### 3.3. Additional Modifiers of Migration Invasion

#### 3.3.1. TRPM

TRPM7 is notable for its differential regulation of glioma behavior through two distinct modalities. While its intrinsic kinase activity was shown to be important for cell migration and invasion, its channel activity influenced cell growth (Table 2) [94]. TRPM8 stimulation with agonists menthol or icilin was sufficient to increase the rate of GBM cell migration via enhanced Ca^2+^ influx [97,100]. RNAi knockdown of *TRPM8* reduced GBM migration speed and transfilter chemotaxis [97]. TRPM8-mediated Ca^2+^ entry has been postulated to activate Ca^2+^-activated K^+^ ion channels (BK channels), thereby driving migration through trailing edge cell shrinkage [91,100]. Support for TRPM8-mediated BK activation comes from a study that found activation of TRPM8 increased the open probability of BK channels [100].

#### 3.3.2. TRPV4

TRPV4 increased GBM migration and invasiveness [104]. Stimulation of TRPV4 with agonist GSK1016790 promoted glioma cell migration, similar to what has been found in breast cancer migration [104]. TRPV4 migration was mediated by phosphorylation of Akt and activation of Rac1, which was prevented when TRPV4 was blocked by antagonist HC-067047 [104]. These proteins are important for cytoskeletal remodeling and cell adhesion.

#### 3.3.3. ASIC1

Acid-sensitive ion channel 1 (ASIC1) was transiently activated by extracellular acidosis. Sheng and colleagues found that ASIC1 was functionally expressed in U87MG and A172 glioma cells [107]. Treatment with a weak acid was sufficient to increase the migration of these cell lines. Conversely, blocking ASIC1b activity with PcTx1 (psalmotoxin1—found in the venom of the west indies tarantula) or reducing ASIC1 expression through shRNA knockdown reduced migration of glioma cell lines [107,108]. Together this indicated ASIC channels promoted glioma cell migration. High-grade gliomas were found to express additional ENaC/Degenerin family members: ASIC, αENaC, and γENaC. It is thought that they combine to form an amiloride-sensitive nonselective cation channel. RNAi knockdown of any of these three channels was sufficient to prevent cell migration potentially through the prevention of Na^+^ influx-mediated lamellipodium expansion [109,110].

#### 3.3.4. PIEZO

A large RNAseq study of 1633 glioma samples determined that PIEZO1 expression was highly correlated with malignant GBM and poor survival outcomes [24]. Assessing PIEZO gene ontology identified an expression correlation with proteins involved in ECM organization and cell migration [24]. The Huang lab found that PIEZO was important for glioma mechanotransduction-mediated progression. Here it mediated multiple ECM remodeling signaling pathways [23]. More specifically, they found that PIEZO1 localizes to focal adhesions where it activated integrin-FAK signaling to regulate the ECM and reinforced tissue stiffening **(**Figure 2C). Their study concluded that the stiffer mechanical microenvironment found in fly and mouse glioma models activated the mechanotransduction channel, PIEZO1, to promote glioma aggression [23]. Interestingly, PIEZO is implicated in the migration of other cancer and non-cancer cell types [184]. It has also been implicated in the amoeboid style of movement in *Dictyostelium* cells [179]. *Dictyostelium* is a single-cell social amoeboid that tends to move via pseudopods, a cellular projection which involves actin polymerization at their leading edge when moving under a buffer. However, if they are exposed to a stiff agarose overlay, termed a “cell squasher”, they transition their movement to PIEZO-dependent blebbing driven motility [179]. In contrast, studies in breast cancer cells found that activation of PIEZO in response to pressure or applied agonist Yoda1 repressed breast cancer cell blebbing [178]. Thus, the role of PIEZO in blebbing and amoeboid movement is still emerging, and these studies were performed in very different cell types and conditions. Future work is needed to determine if PIEZO might mediate the transition between modes of migration in the rounded amoeboid moving cells found in glioma 3D cultures in response to the changing cellular environment [177].

#### 3.3.5. NHE

Na^+^/H^+^ exchangers (NHE1-9) are major regulators of intracellular pH. In recent years, studies have shown that the extracellular environment near tumors is acidified, known as extracellular acidosis, with pH values ranging from 6.1 to 6.8 [185,186]. An acidic extracellular pH (pHe) in the tumor microenvironment has been suggested to promote glioma invasion by inducing cytoskeletal rearrangement, protrusion, epithelial–mesenchymal transition (EMT), and degradation of the extracellular matrix [187]. Studies have detected upregulated protein and mRNA (*SLC9A1*) expression of NHE1 in primary human glioma cells, glioma xenografts, and glioblastomas [28]. NHE1 localizes to the plasma membrane of most cells. Proximity ligation assay (PLA) experiments have implied that NHE1 activates matrix metalloproteinases (MMPs), a family of endopeptidases that cleave most extracellular matrix constituents, by providing an ideal extracellular acidic environment for its catalytic activity [28,188]. Kurata et al. identified a role for NHE5 in glioma progression [21]. NHE5 cycles between recycling endosomes and the plasma membrane, where it regulates the pH of the cytosolic and endosomal lumen. NHE5 was not found in astrocytes but was upregulated in C6 glioma cells [21]. Moreover, the knockdown of NHE5 reduced growth signaling of both MET and EGFR and limited cellular spreading. Reducing NHE5 expression reduced endosomal recycling and protein stability of β-1 integrin (Figure 2B) [21]. Together, this suggested that NHE5-mediated protein trafficking is important for regulating cell adhesion and promoting glioma invasion.

### 3.4. Kir4.1—Inhibitor of Cellular Migration

*Kir4.1 (KCNJ10)* is expressed in glial cells but tends to be absent or mislocalized in gliomas [31,32]. Thuringer and colleagues identified that the microRNA, MiR-5096 inhibited Kir4.1, which may explain why Kir4.1 expression levels are decreased in gliomas. Kir4.1 in astrocytes was important for promoting differentiation [189]. Gliomas may downregulate Kir to promote their growth and migration. Evidence for this is supported by work by Higashimori et al., who found ectopic expression of Kir4.1 reduced glioma growth (D54-MG cells) [48]. Further reduction of Kir4.1 in gliomas through loading cells with miR-5096 or channel blocker barium inhibition led to increased formation of filipodia and invasiveness of U87MG and U251 glioma cell lines [32]. miR-0596 was also sufficient to increase the release of extracellular vesicles that contained miR-5096 in a Kir4.1-dependent way. This could provide gliomas with a mechanism to disseminate suppression of Kir4.1 to promote glioma growth and invasiveness while compromising the survival of non-tumor glial cells.

### 3.5. Tumor Microenvironment (TME) influence on Migration and Invasion

Venkatarami and colleagues found that neural-glioma synapses are functionally active. Neural-glioma synapses exhibited Ca^2+^ activity in response to neural activity which in turn promoted invasiveness [42,127]. Tumor migration was positively correlated with the frequency of Ca^2+^ signals. Knocking down AMPAR signaling led to a reduction in GBM cell invasion, indicating it was an important mediator of neuronal-driven GBM cell behavior [42].

## 4. Glioma Metabolism

**Figure 3:**
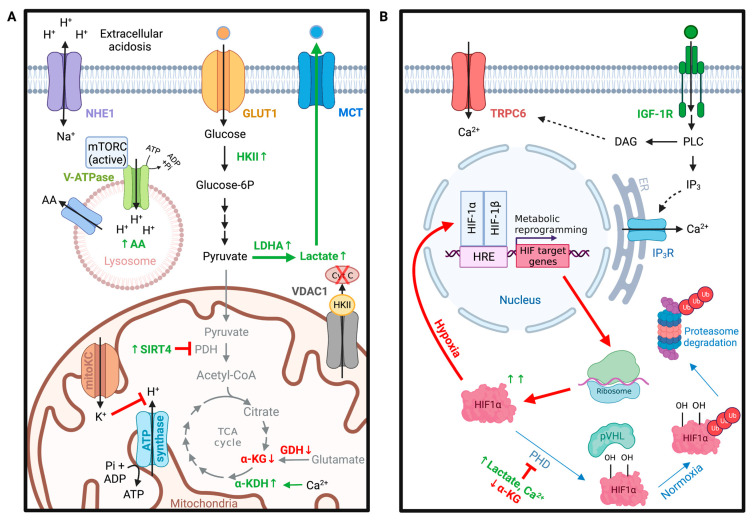
Ion channels in glioma metabolism. (**A**) Ion channels involved in glioma metabolic shift toward aerobic glycolysis over mitochondrial oxidative phosphorylation (Warburg effect). (**B**) TRPC6 channels control the stability of HIF1α in gliomas under hypoxia resulting in the upregulation of genes involved in glioma’s metabolic reprogramming. Abbreviations: α-KDH = α-ketodehydrogenase, α-KG = α-ketoglutarate, AA = amino acid, Cyt C = cytochrome c, GDH = glutamate dehydrogenase, GLUT1 = glucose transporter protein type 1, HKII = hexokinase II, Kv1.3 = voltage-gated potassium channel, LDHA = lactate dehydrogenase A, MCT = monocarboxylate transporter, mitoKC = mitochondria K^+^ channels, mTORC = mammalian target of rapamycin, NHE1 = Na-H exchanger 1, PDH = pyruvate dehydrogenase, SIRT4 = sirtuin 4, TCA cycle = tricarboxylic acid cycle, V-ATPase = vacuolar-type ATPase, VDAC1 = voltage-dependent anion channel 1. DAG = diacyl glycerol, ER = endoplasmic reticulum, HIF1α = hypoxia-inducible factor 1 α, HRE = hypoxia-response elements, IGF-1R = insulin-like growth factor 1 receptor, IP_3_ = inositol 1,4,5-triphosphate, IP_3_R = inositol 1,4,5-triphosphate receptor, PHD = prolyl hydroxylases, PLC = phospholipase C, pVHL = von Hippel-Lindau tumor suppressor, TRPC6 = transient receptor potential cation channel subfamily C member 6. Created with BioRender.com.

All cells require nutrient uptake from their surrounding environment to grow and divide. The mammalian brain relies on glucose as its primary source of energy, utilizing nearly half of the body’s daily glucose intake and 20% of the body’s oxygen [190]. Astrocytes and oligodendrocytes rely on glycolytic metabolism, the cytosolic pathway of glucose breakdown, whereupon one molecule of glucose is broken down into two molecules of pyruvate, along with a net production of two molecules of ATP and NADH [191,192]. In neurons, microglia, and some glioma cancer stem cells, oxidative metabolism predominates [193,194,195]. Here, the pyruvate produced from the glycolytic breakdown of glucose enters the mitochondria, where it is converted to acetyl-CoA and further metabolized in the mitochondrial tricarboxylic acid cycle (TCA cycle). The TCA cycle transforms the chemical energy from acetyl-CoA into the reducing power of NADH, which subsequently generates electrons for the electron transport chain that produces ATP. The glycolytic pathway is highly upregulated in gliomas, favoring glucose breakdown into lactate despite sufficient oxygen levels. This characteristic aerobic glycolysis is a hallmark of cancer known as the Warburg effect (Figure 3A) [196]. 

It is hypothesized that this metabolic reprogramming allows carbons derived from glucose to be utilized for the synthesis of essential cellular components while regenerating NAD^+^ from NADH, an oxidizing agent required for the continuation of glycolysis [197]. Furthermore, glioblastomas display differential expression levels of glutamate metabolism modulators [198]. Hexokinase II (HKII), lactate dehydrogenase A (LDHA), sirtuin 4 (SIRT4), and glutamine synthetase (GS) have been shown to be elevated in glioblastomas [199,200,201]. In contrast, glutamate dehydrogenase (GDH) protein expression levels have been shown to be reduced in U87MG glioblastoma cell lines compared to human astrocyte controls [201]. These metabolic adaptations appear to be responsive not only to the tumor’s microenvironment but also to the tumor’s genotype. Recent studies increasingly describe alterations of ion channel expression that correlate to changes in glioma cell metabolism. However, the molecular basis for ion channel regulation in glioma-related metabolic reprogramming remains elusive. Open questions include: (1) Which ion channels regulate metabolic homeostasis of gliomas? (2) What roles do ion channels have in the pH regulation of glioma glycolytic-driven metabolism? (3) How do ion channels support hypoxia-induced metabolic shifts in gliomas?

### 4.1. VDAC in Metabolic Homeostasis of Gliomas

Mitochondria are major hubs for cellular metabolism, oxidative stress, and apoptosis. Approximately 32 ATP molecules are generated per molecule of glucose during oxidative phosphorylation within the mitochondrial matrix [202]. This is in stark contrast to only 2 ATP molecules generated during anaerobic glycolysis [202]. In gliomas, mitochondrial function is impaired. This metabolic shift in glioma cells results in compromised utilization of the oxidative phosphorylation pathway with a resultant increase in glycolytic metabolism [203]. One of the most abundant channels in the mitochondrial outer membrane is the voltage-dependent anion channel 1 (VDAC1) [204]. VDAC1 is highly expressed in glioblastomas, exhibiting its significance in high energy-demanding cancer cells [111]. As several organic anions, respiratory substrates, and metabolites must pass through VDAC1 to enter or exit the mitochondria, VDAC1 is crucial for regulating cellular energy and metabolic homeostasis [111]. As the name implies, VDAC1 is voltage-dependent. During depolarizing potentials (approximately −40 to +40 mV), VDAC1 exhibits an open conformation that is highly favorable to organic anion permeability as well as ATP, ADP, inorganic phosphate (Pi), and various metabolites [204]. At higher positive or negative potentials, VDAC1 exhibits a closed conformation that favors the transport of cations (e.g., K^+^, Na^+^, Ca^2+^) and is impermeable to ATP [113]. VDAC1 thus acts as a switch that can have pro-Warburg effects (increased aerobic glycolysis, closed conformation) or turn on mitochondrial metabolism (anti-Warburg, open conformation). Studies have shown that the reduction of VDAC1 in patient-derived glioblastoma cell lines by way of si-RNA reduced cellular ATP levels and inhibited tumor development and growth [111]. VDAC2 similarly regulated glucose metabolic reprogramming of glioma stem cells by directly binding phosphofructokinase (PFKP) and preventing its release into the cytoplasm to regulate glycolysis [114].

Although the shift to aerobic glycolysis in gliomas caused by the closure of VDAC1 is less efficient for ATP production, the shift enhances the cancer cells’ resistance to apoptosis. Along with regulating cellular metabolism, VDAC1 also plays a key role in mitochondrial-mediated apoptosis by interacting with both pro- and anti-apoptotic factors (i.e., hexokinase, Bcl2, Bcl-xL) and allowing passage of apoptotic factors (i.e., cytochrome c and apoptosis-inducing factor (AIF)) [111,113]. HKII is of particular interest as it is known to be elevated in gliomas and a key player in cancer cell survival. HKII increasingly translocated to the outer mitochondrial membrane, where it was associated with VDAC1. VDAC-bound HKII has been shown to be more resistant to apoptosis by preventing cytochrome c release [205].

### 4.2. K^+^ Channels in Glioma Metabolism

Glycolytic enzymes require monovalent cations such as Ca^2+^, Mg^2+^, and Zn^2+^ to activate their catalytic centers [206]. One of the most noteworthy examples of a metabolic enzyme that requires K^+^, Mg^2+^, and Mn^2+^ for its activity is pyruvate kinase (PK) [206]. The orchestrated presence of these cations enables substrate binding to the active site. K^+^ is distinctly critical as it facilitates the transfer of phosphate groups from the substrate to ATP [207]. Recent studies have additionally identified HKII as a K^+^-dependent glycolytic enzyme [208]. Intracellular K^+^ ions exert stabilizing effects on HKII, and the reduction of intracellular K^+^ ion levels notably disrupts HKII-dependent glycolysis [208]. Considering that several key glycolytic enzymes require K^+^ as a cofactor for proper function, dysregulated K^+^ channel expression could potentially be a therapeutic target for glioma cells with altered metabolic programming. However, while numerous studies have shown that various K^+^ channels are differentially expressed in glioma cells and are involved in cell cycle regulation, K^+^ channel involvement in glioma metabolism is less known. Most K^+^ channels are found at the plasma membrane, but there are also nine different K^+^ channels localized in the intermembrane space of the mitochondria: mitoKATP, mitoBKca, -IKca, SKca, mitoHCN, mitoKv1.3, Kv7.4, mitoTASK-3, and finally mitoSLO2 [209,210,211]. Opening of these mitochondrial K^+^ channels permits K^+^ influx into the mitochondrial matrix. The roles of each of these channels are diverse, but the general K^+^ influx into the mitochondrial matrix could potentially inhibit ATP Synthase activity by disrupting the mitochondrial membrane potential [212]. Of the mitochondrial K^+^ channels, large-conductance Ca^2+^-activated K^+^ channels (mitoBKCa) are of particular interest because they are highly expressed in human glioma cells [33,73,213,214]. Activation of mitoBKCa using BKCa-channel openers (CGS7181 and CGS7184) in a human glioma cell line (LN229) triggered cell death of human glioma cells through increased cytosolic calcium concentrations required for calcium-activated proteases [33]. MitoKv1.3, on the other hand, was required for glioma apoptosis through Bax-dependent mitochondrial pathways, and human cells genetically deficient in Kv1.3 resisted apoptosis [73].

### 4.3. Role of pH in Glioma Metabolism

Intracellular pH (pHi) is critical for the regulation of metabolism, as an alkaline pHi is one of the main driving forces for glycolytic metabolism [215]. The extracellular pH (pHe) of glioma cell populations can fall to values below 6.5 due to extracellular acidosis and the generation of lactate [216]. Interestingly, the pHi measured within tumors is akin to or even slightly more alkaline compared to normal cells [217]. As an acidic pH is detrimental to cellular function, the build-up of protons within the cell must be pumped out to maintain proper cell function. Cancer cells utilize several proton transporters to execute acid extrusion, including Na^+/^H^+^ exchanger 1 (NHE1), a major regulator of pHi [28]. NHE1, encoded by the *SLC9A1* gene, is known to be localized at the plasma membrane and functions by mediating H^+^ efflux in exchange for Na^+^ influx [218]. Recent studies have documented that NHE1 is upregulated in human glioblastoma cells and plays a role in glucose metabolism dysregulation in glioblastomas. [29,30]. The reversed pH gradient across the plasma membrane of glioma cells where pHe is more acidic than pHi presents a highly malignant and invasive phenotype that promotes glycolytic metabolism, which in turn supports a highly malignant and proliferative phenotype. Pharmacological blockade of NHE1 by NHE1-specific inhibitor HOE642 in combination with the chemotherapeutic drug temozolomide in mouse-derived glioma-associated microglia/myeloid cells significantly restored oxidative phosphorylation and reduced glycolysis [115]. Similarly, administering HOE642 to a mouse glioma model resulted in reduced glioma volume and malignancy [28]. This provides some prospects for the future development of metabolic modulators used as adjuvants with chemotherapeutic and radiation therapies.

Another proton pump that cancer cells use to execute acid extrusion is the vacuolar H^+^-ATPase (V-ATPase) [219]. V-ATPase is made up of multiple subunits and isoforms arranged in two domains: a peripheral V1 domain, responsible for ATP hydrolysis, and an integral membrane domain V0, which functions in proton translocation. The V1G1 subunit encoded by *ATP6V1G1* was upregulated in glioblastoma patient-derived neurospheres [220]. V-ATPases are mainly localized on the surface of endosomes but can also be found on the plasma membrane of specialized cell types [221]. They harness the energy released from ATP hydrolysis and transport protons from the cytosol to either the lumen of endosomal compartments or into the extracellular space. V-ATPases are essential for cellular nutrient and energy homeostasis [222]. The acidic pH of lysosomes is necessary for proper proteolytic enzymatic function, and the proton gradient generated from V-ATPase additionally drives the coupled export of amino acids into the cytoplasm to be recycled into the building blocks of cellular machinery. Furthermore, V-ATPase is known to be required for the recruitment of the metabolic regulators—mTORC1 (mechanistic target of rapamycin complex 1) and AMPK (AMP-activated protein kinase) [78]. The primary method of regulating V-ATPase activity in vivo is by way of reversible assembly/disassembly of the ATP-hydrolytic V1 domain form the proton-translocating V0 domain. Assembly occurs in response to elevated glucose concentrations, amino acid starvation, and exposure to growth factors. This increased assembly permits expulsion of metabolic acid generated by increased glycolysis.

### 4.4. TRPC Channels in Hypoxia-Induced Metabolic Shifts in Gliomas

Hypoxia plays a major role in the malignancy and aggressiveness of glioblastomas [223]. This is largely a reflection of the rapid proliferation of tumor cells, causing some tumor cells to be located further away from the oxygen-providing vascular system. Cells subjected to hypoxia activate several sets of complex responses to alter their metabolism to temporarily escape nutrient deprivation and cell death. Furthermore, hypoxic regions of brain tumors have been documented to display the most aggressive behavior, and it is in these regions where cells drive tumorigenesis and manifest resistance to most treatments [224]. As oxygen is the final electron acceptor in the electron transport chain, the hypoxic environment reduces aerobic oxidative respiration and instead generates reactive oxygen species (ROS). Hypoxia-inducible factor 1 (HIF-1), an oxygen-sensing heterodimeric transcription factor, functions as a master regulator of oxygen homeostasis and is upregulated in tissues adjacent to glioma necroses [225]. Its stability is primarily controlled by prolyl hydroxylation via prolyl hydroxylases (PHDs) of its oxygen-dependent degradation domain (ODDD) in its alpha subunit, HIF-1α [226]. This reaction requires α-ketoglutarate, O_2_, and Fe^2+^ as substrates and can be competitively inhibited by succinate, fumarate, and 2-hydroxyglutarate [227]. Recent reports have found that hypoxia in glioma cells led to the activation of transient receptor potential canonical 6 (TRPC6) channels by the IGF-1R-PLCγ-IP_3_R pathway (Figure 3B) [228]. Studies performed in U251MG and U87MG glioma cell lines revealed that TRPC6 influenced the stability of HIF-1α by controlling its hydroxylation [228]. Inhibition of TRPC6 not only suppressed the influx of intracellular Ca^2+^ but also increased α-ketoglutarate levels and promoted PHD activities, all of which promoted HIF-1α hydroxylation to suppress HIF-1α accumulation. As one of HIF-1α’s target genes is glucose transporter I (GLUT1), inhibition of TRPC6 reduced glucose uptake during hypoxia, thus exhibiting its role as a significant metabolic regulator of glioblastomas.

## 5. Blood–Brain Barrier, Microenvironment, and Angiogenesis

This section reviews the current state and new developments in three important areas related to the pathophysiology of GBM: blood–brain barrier, microenvironment, and angiogenesis. Despite the substantial overlap, each field is frequently approached with different conceptual and methodological premises. As we detail below, the roles of ion channels in all three of these fields of brain cancer research provide an opportunity to highlight both unique and overlapping aspects related to the pathophysiology of central nervous system neoplasia. Understanding their interrelatedness also provides important entry points for the development of novel therapeutic strategies aimed at enhancing the quality of life of affected patients. Some of these novel therapies utilize enhanced penetration of drugs into tumors [229], in vitro-engineered artificial blood vessels, and antiangiogenic therapy.

### 5.1. Blood–Brain Barrier in Brain Tumors

The blood–brain barrier (BBB) is an essential cellular boundary that controls the microenvironment of the central nervous system (CNS) by regulating the passage of molecules into and out of the CNS to maintain a physiological environment required for normal neuronal functioning [230]. Maintenance of the functional and structural integrity of the BBB ensures a homeostatic brain microenvironment, whereas its disturbance through invasive brain tumors creates a favorable environment for malignant proliferation, treatment resistance, and secondary complications.

**Figure 4:**
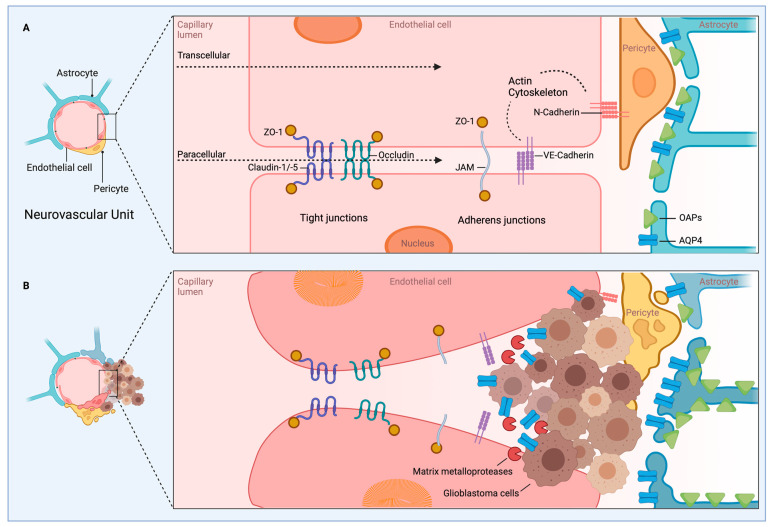
Neurovascular unit (NVU) and blood–brain barrier (BBB) in glioblastoma. (**A**) The NVU provides the structural correlation of the BBB. At the capillary level, the NVU is comprised of endothelial cells connected through tight junctions (TJ) in spatial arrangement with adherens junctions (AJ), the basement membrane (not shown), pericytes, microglia (not shown), and the astrocyte endfeet. Central to the barrier function of the BBB is TJ, with its major components being claudin-1, claudin-5, and occludin. Expression of TJ is coregulated with AJ, whose main building blocks are cadherin (shown here are VE-cadherin and N-cadherin), which connect to the actin cytoskeleton via catenins (not shown). The paracellular transport pathway is closely related to the barrier function of the BBB and regulated by changes in the permeability of the TJ/AJ proteins connected to the actin cytoskeleton. In contrast, the transcellular transport pathway has a major role in the transport function of the BBB and is mediated mainly through pericytes and endothelial cells via several mechanisms, including passive diffusion, active efflux, carrier-mediated transport, receptor-mediated transport, and immune cell function. Aquaporin 4 (AQP4), the main water channel in the brain, is primarily found at perivascular astrocytic endfeet. AQP4 helps form the highly structured orthogonal arrays of intramembranous particles (OAPs) located at the interface of astrocytic endfeet and the superficial and perivascular basal lamina, which serve as a critical functional component of the BBB. (**B**) Pathological remodeling of the BBB. Glioblastoma causes structural and functional alterations in the BBB, leading to a disruption of normal brain architecture and molecular interactions. The structural proximity between pericytes and endothelial cells becomes increasingly dissolved in glioma tissue. This leads to impaired integrity of the BBB and a loss of its transport function, which contributes to chronic hyperpermeability, fluid accumulation, and hypoxia and facilitates metastatic invasion. Astrocytes undergo reactive changes in response to glioblastoma, which in return contributes to a microenvironment that is conducive to migration, proliferation, and invasion of glioblastoma. Invasion involves the separation of astrocytic end feet away from the basement membrane of endothelial cells, followed by degradation of the basement membrane and loss of tight junctions, eventually leading to intravascular fluid leakage into the surrounding parenchyma. Glioma cells show dysregulated expression and disordered localization of AQP4, which is expressed as a non-OAP-associated form throughout the astrocytic membrane rather than restricted to the OAPs in the glial endfoot membranes. This contributes to the breakdown of the BBB in glioma resulting in increased water permeability and vasogenic brain edema. Created with BioRender.com.

Here, we briefly summarize the cellular and structural players most relevant to this review, along with a high-level overview of the physiological BBB functions at the brain–blood interface. In the strict sense, the BBB is created by the endothelial cells lining the cerebral capillaries that penetrate the brain and spinal cord [231]. Endothelial cells of the BBB are unique because they have continuous tight junctions (TJs), lack fenestrations, and essentially have no pinocytosis [232]. As such, the BBB is the heart of a cellular organization referred to as the neurovascular unit (NVU). The NVU is comprised of the capillary endothelium connected through TJs, the basement membrane, the pericytes, the microglia, and the astrocyte endfeet (Figure 4A). The core functions of the BBB can be divided into barrier and transport functions. Central to the barrier are endothelial tight junctions (TJ), multiprotein complexes that tightly isolate paracellular passages between adjacent endothelial cells. Endothelial TJs are similar to epithelial TJs but more complex in that they are coregulated with adherent junctions (AJs), with which they form the extracellular junction complex essential for the barrier function of the BBB [233]. TJs are mainly made up of claudins, with claudin-1, claudin-5 and occludin being the major sealing proteins in the BBB [233,234,235]. The main building blocks of adherens junctions (AJs) are cadherins, which connect to the actin cytoskeleton via catenins to form adhesive contacts between cells. The expression of TJs and AJs in BBB endothelial cells is coregulated, with high expression of TJ proteins occludin and claudin-5 and low expression of AJ protein VE-cadherin [236]. For a more in-depth review of the interrelationship of junctional complexes, cytoskeleton structure, and the signaling pathways involved, please see [236,237].

The endothelial junctional complex of the BBB controls the paracellular transport pathway, which is closely related to the barrier function of the BBB and regulated by changes in the permeability of the TJ/AJ/occludin proteins connected to the actin cytoskeleton. In contrast, the transcellular transport pathway has a major role in the transport function of the BBB and is conveyed mainly through pericytes and endothelial cells via several mechanisms, including passive diffusion, active efflux, carrier-mediated transport, receptor-mediated transport, and immune cell function [238]. The complex expression patterns of countless transporters, receptors, active efflux pumps, ion channels, and regulatory molecules studied by BBB cell type and location have recently been reviewed as well [239]. For the purpose of this review, we will focus on ion channels and transporters involved in the BBB, which will be discussed below.

The pathogenesis of invasive brain tumors is fundamentally linked to structural and functional alterations in the BBB, leading to a disruption of normal brain architecture and molecular interactions (Figure 4B). For brain tumors, the pathophysiology underlying these changes has been studied most extensively in glioblastomas. This process is also described as remodeling of the BBB. Here, we will only provide a brief overview of some of the structural changes of BBB remodeling and its effects on glioma and then will focus on cellular and molecular changes related to ion channels in the BBB this process. Remodeling of the BBB is driven by the local effects of glioma cells on their immediate environment and remote effects mediated by secreted molecules. Invasion of glioma typically proceeds along pre-existing structures in the brain, such as blood vessels, meningeal layers, and white matter fibers [174].

Changes in blood vessels related to glioblastoma include modification of existing blood vessels and promotion of angiogenesis, which is reviewed below as it relates to ion channel involvement (for a general review, see [240]). Both pericytes and astrocytes are involved in the pathological remodeling of the BBB barrier. The structural proximity between pericytes and endothelial cells becomes increasingly dissolved in glioma tissue (Figure 4B). The consequence for the BBB barrier is an impaired integrity of its barrier function and a loss of its transport function, which contributes to chronic hyperpermeability, fluid accumulation, and hypoxia and facilitates infiltrative behavior. Astrocytes also undergo characteristic reactive changes in response to glioblastoma, which in return contributes to a microenvironment that is conducive to migration, proliferation, and invasion of glioblastoma. Invasion involves the separation of astrocytic end feet away from the basement membrane of endothelial cells and into the abluminal side of vessels, followed by degradation of the basement membrane and loss of tight junctions, eventually leading to intravascular fluid leakage into the surrounding parenchyma. The concept of reactive astrocytes and their role in astrocyte-glioblastoma crosstalk in the pathophysiology of glioblastoma have been reviewed in detail [241]. Damage of the BBB leads to extravasation of fluid and brain edema, tumor growth, and tumor invasion. Conversely, glioma cells stimulate vascular endothelial cells to proliferate abnormally, supporting tumor neovascularization, which may further accelerate the occurrence and development of glioma.

### 5.2. Overview of BBB Ion Channels and Transporters

On a molecular level, channels, transporters, and receptors in endothelial cells and pericytes, and likely also in astrocytes, tightly regulate the exchange between blood and the brain primarily through transcellular transport mechanisms. We include a brief overview of physiological ion channel transporters before focusing on those channels shown to be involved in BBB remodeling related to glioma. Ion transporters in BBB endothelial cells involved in regulating ion concentrations in the brain include sodium and chloride ion transporters (Na^+^/K^+^-ATPase, luminal Na^+^/K^+^/Cl^−^ cotransporter (NKCC1)), HCO_3_^−^/Cl^−^ exchanger, luminal Na^+^/H^+^ exchanger, calcium transporters (Na^+^/Ca^2+^ exchanger cotransporter, TRP channels) and potassium channels (voltage-gated K^+^ channel KV1, inward rectifier K channel KIR) [49,50,51,52,53,54,55,56,57]. Ion transporters expressed in BBB pericytes include Na^+^/K^+^-ATPase, Ca^2+^ ATPases, Na^+^/K^+^/Ca^2+^ exchanger SLC24A3, Na^+^/H^+^ exchanger SLC9A3R1, Cl-HCO_3_ exchanger SLC4A4 and SLC4A3, Na^+^/Ca^2+^ exchanger SLC8A2, ATP-sensitive K^+^ channel ATP-binding cassette ABCC9, H^+^-peptide transporter SLC15A2, Na^+^/I^−^ symporter SLC5A5, and inwardly rectifying potassium (KIR) channel Kir6.1 [71,72,74,76,79,242]. The contribution of astrocytes to BBB function via the polar distribution of water channels and astrocytic endfeet is reviewed below.

### 5.3. Aquaporins

Aquaporins (AQPs) are transmembrane proteins responsible for fast bidirectional water movement across cell membranes, and some also transport other small molecules, such as glycerol and urea. In addition to contributing to BBB integrity, they serve diverse physiological functions including water homeostasis [243,244], brain metabolism and microenvironment [245], neuronal formation [246], proliferation, migration and neuronal differentiation [247,248], communication with neurons, and synaptogenesis [249]. The AQP subtypes expressed in astrocytes include AQP1, AQP3, AQP4, AQP5, AQP8 and AQP9, and their expression profiles and subcellular localizations correlate with both their physiological and pathophysiological functions [250].

Expression studies in human astrocytes have documented upregulation of AQPs in numerous pathological states, including neurodegenerative diseases, edematous brain tumors (notably, in brain edema, both up and downregulation have been reported), peritumoral tissue, ischemia, and subarachnoid hemorrhage [34,251,252,253,254]. The pathophysiological roles of this group of transporters in glioma development are supported by their expression profiles [34,255,256,257]. Despite having been linked to various aspects of glioma pathogenesis, including infiltration [258], brain edema [259], migration [248], hypoxia-induced invasion of glioma [260] (also demonstrated for neuroblastoma [261]), and metabolic adaption of glioma cells to glycolysis [262], the specific functions of individual AQP subtypes are in many cases incompletely understood. AQP1 and AQP4 have been studied most extensively in glioma. AQP4, the main subtype shown to contribute to BBB remodeling and brain edema in glioma, is discussed in further detail below. Transcriptional and protein expression studies in a human brain specimen showed that AQP1 is overexpressed in astrocytomas, and there is emerging evidence for a correlation between AQP1 expression levels and histological grades of astrocytoma with glioblastoma showing the highest AQP1 expression [34,35,36]. This upregulation is found in perivascular areas of tumor infiltration, indicating involvement in angiogenesis and invasive behavior and providing a rationale for potential treatment approaches [36]. Upregulation of AQP1 has also been related to glioma cell migration [123]. Limited expression data indicate the possible involvement of AQP5 and AQP8 in glioma development, whereas no data exist for the other AQP subtypes. With regard to brain pathophysiology, the functions of AQPs have been worked out most extensively for brain edema, with most of the evidence coming from studies of the AQP4 subtype.

Aquaporin 4 (AQP4), the main water channel in the brain, is arranged in a characteristic asymmetric distribution within the astrocyte membrane where it is primarily found at the end of astrocytes in the vicinity of microvessels as part of a functionally important cell polarity also referred to as perivascular astrocytic endfeet (Figure 4A). This protein helps form the highly structured orthogonal arrays of intramembranous particles (OAPs) located at the interface of astrocytic endfeet and the superficial and perivascular basal lamina, which serve as a critical functional component of the BBB and are dependent on the extracellular matrix component agrin for correct assembly and localization [263]. Glioma cells show dysregulated expression and disordered localization of AQP4, which is expressed as a non-OAP-associated form throughout the astrocytic membrane rather than restricted to the OAPs in the glial endfoot membranes (Figure 4B). This contributes to the breakdown of the BBB in glioma resulting in increased water permeability and vasogenic brain edema. The redistribution of AQP4 appears to be mediated in part by changes of dystroglycan and agrin complexes by matrix metalloproteinases (MMP) 2, 3, and 9 and also affects water flow through AQP4 channels, further contributing to brain edema [37,38]. Vascular endothelial growth factor (VEGF), known to induce BBB permeability and disruption, likely in part by downregulation of claudin-5 and occludin, has also been shown to upregulate AQP4 expression, which may in part be a reaction to vasogenic edema induced by VEGF [264,265,266].

Mouse models recapitulate the diverse roles of AQP4 in glioma-related brain edema, although the phenotypes produced in knockouts have been variable. Zhou et al. observed the loss of astrocyte cellular polarity, altered microvessel ultrastructure, leaky tight junctions, astrocyte swelling around blood vessels, hyperpermeability of the BBB, and brain edema in AQP4 knockout mice. [267]. The absence of AQP4 resulted in increased capillary density and decreased water exchange in the BBB in the mice reported by Zhang et al. [268]. In contrast, Saadoun et al. did not find changes in BBB integrity or brain morphology in mice lacking AQP4 [269]. Further research is needed to clarify these diverging observations.

In line with the evolving model of aquaporin function in glioma-induced brain edema are mRNA and protein expression studies in human brain tumor specimens that show the highest expression levels of AQP4 in pilocytic astrocytomas WHO grade I and grade IV glioblastomas, both tumor types with a high degree of BBB remodeling and angiogenesis, with a progressive increase in expression levels from WHO grade II to IV [270]. These and other observations [271] have raised interest in exploring aquaporins as diagnostic and prognostic biomarkers in brain cancer and other CNS pathologies [272,273]. Similarly, peritumoral expression levels have been shown to correspond positively with the degree of peritumoral edema and histological grade as determined by brain MRI studies and pathological examination [253]. The differential expression of aquaporins related to pathophysiological CNS processes has been exploited to develop targeted therapy approaches using micro-RNAs [274,275,276].

Although not strictly regarded as ion channels, it is worth briefly considering gap junction proteins at this juncture as they display close proximity to AQP4 [277] and expression of connexin 43 (Cx43), the most widely studied gap junction protein involved in the diffusion of water and solutes within the astrocytic network appears to be affected by AQP4 expression [278]. Gap junction (GJ) channels and their connexins (Cx) are central to cell communication in the CNS and are highly expressed in neurons, astrocytes, oligodendrocytes, and microglial cells. Cx43 has been shown to be overexpressed in glioblastoma. While some studies have reported an inverse correlation between Cx43 and tumor grade, the significance of these findings remains unclear [279]. In addition to contributing to the regulation of genes related to cell cycle control and, as such, acting as a tumor suppressor, a central role for Cx43 is emerging in cancer cell motility and glioblastoma cell migration. It has been proposed that the seemingly opposing roles in gliomagenesis, tumor suppressor function, and promotion of cell migration by Cx43 may be reflecting different stages in cancer formation as migrating cells are usually not proliferating at the same time and vice versa [280]. It has been known for some time that gap junction proteins can serve as connections for glioblastoma cells (GBCs) organizing into tumor cell networks. These networks appear to be central in mediating the characteristic treatment resistance of glioblastoma [176]. More recent studies indicate that gap junction may not only connect GBCs among themselves but also mediate connections between GBCs and astrocytes, widening the concept of the tumor network in glioblastoma [42]. 

### 5.4. Microenvironment in Brain Tumors

**Figure 5:**
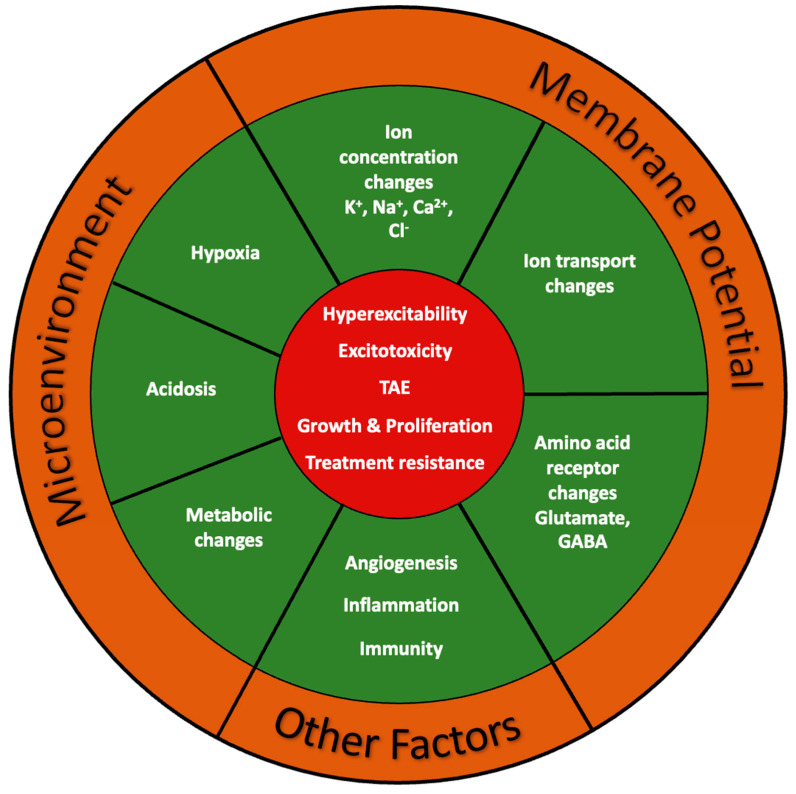
Microenvironment in brain tumors. The interactions between cancer cells and tumor microenvironment (TME) are essential determinants of many hallmarks related to cancer development. TAE, tumor-associated epilepsy.

A tumor is not simply a growing cluster of cancer cells. Rather, tumor formation and progression are complex processes that involve genetic and epigenetic changes affecting the tumor cells as well as changes in the tumor’s surroundings through mutual and dynamic crosstalk [281]. The whole of the components and conditions surrounding the tumor has been coined tumor microenvironment (TME). Although variable depending on tumor type, the TME typically consists of immune cells, stromal cells, blood vessels, and extracellular matrix [282] [Anderson 2020]. The significance of the TME lies in its role as a promoter of cancer formation, progression, and maintenance, as well as in its contribution to treatment resistance [283]. Indeed, the multilayered interactions between cancer cells and tumor microenvironment (TME) are now recognized as an essential determinant of many hallmarks related to cancer development [4]. Moreover, the study of the underlying molecular mechanisms may provide future targets for pharmacological intervention. Understanding the biology and underlying molecular mechanisms of the TME may provide future targets for pharmacological intervention [284]. Although the roles of individual ion transporters in the formation and modification of the TME are still poorly understood, it seems beneficial to outline current overlapping research fields in a review of ion transporters in brain tumors (Figure 5). Given that the molecular exchanges across membranes can both alter the environmental conditions while also being regulated by environmental factors, the TME should always be considered when interpreting experimental data related to ion channel function in central nervous system neoplasia. Recent studies have started to elucidate how the TME is shaped by the diverse routes of communications utilized by glioma cells to impose altered behavior on surrounding non-tumor brain cells. Components of the TME include secreted molecules (chemokines, cytokines), extracellular matrix components (collagen, glycoproteins), extracellular vesicles, tunneling nanotubes, local cell types (neurons, astrocytes, endothelial cells, pericytes, and fibroblasts), and immune cells (microglia, bone marrow-derived macrophages) [176,285,286,287,288]. Neuronal and glial cells reside within the network of the glioma TME. The glioblastoma cell-of-origin hierarchy postulates that neural precursor cells (NPCs) are the principal cell types susceptible to tumorigenesis [289]. NPCs also contribute to the TME in various ways. For example, the release of endovanilloids by NPCs activates the transient receptor potential vanilloid subfamily member-1 (TRPV1) on glioblastoma cells, thereby reducing glioma expansion and prolonging survival time [148].

### 5.5. TME in Tumor-Associated Epilepsy (TAE)

**Figure 6:**
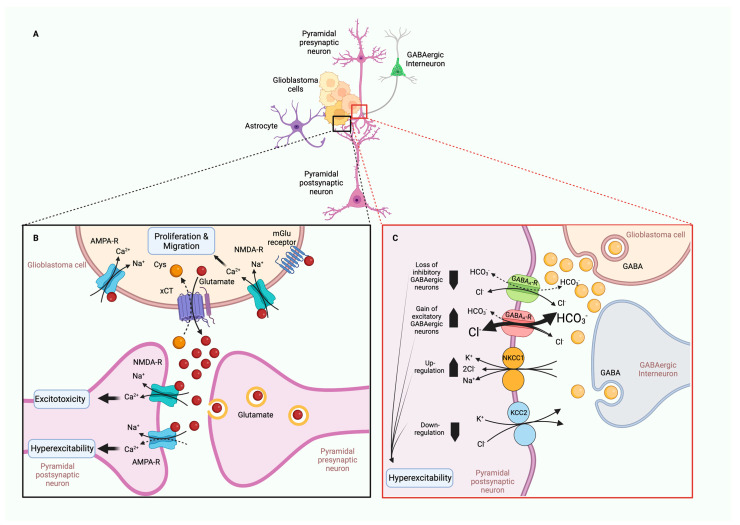
Excitatory and inhibitory networks and their respective neurotransmitter systems also influence the risk of tumor-associated epilepsy (TAE). (**A**) Simplified schematic of glioblastoma cells, cortical neurons, and astrocytes. (**B**) Loss of glutamate homeostasis is a factor in peritumoral seizure activity. Glutamate produced by glioma cells is released in high amounts via the cystine-glutamate transporter (xCT). Binding of glutamate to NMDA and AMPA receptors triggers the opening of cation-specific ion channels that permit influx of Na^+^ or Ca^2+^ ions. Elevated extracellular glutamate promotes excitotoxicity in non-tumor parenchyma and creates excitatory imbalance associated with a higher risk of seizures. (**C**) Loss of GABAergic interneurons in peritumoral tissue combined with a reverse GABAergic effect from inhibitory to excitatory in surviving neurons creates a hyperexcitable tumor microenvironment with increased seizure susceptibility. Downregulation of chloride exporter KCC2 and upregulation of chloride importer NKCC1 leads to excessive intracellular chloride concentrations and causes GABA-mediated depolarization and hyperexcitability. Created with BioRender.com.

Tumor-associated epilepsy (TAE) is a frequent complication in glioma patients. While common in patients with low-grade tumors, patients with high-grade glioblastoma experience seizures less frequently. The pathogenesis of TAE is likely heterogeneous and depends on tumor type, which in turn occurs in typical locations as important determinants for TEA. Within a specific brain tumor histology, additional TAE susceptibility factors include disruption of the physiological BBB, altered expression of gap junctions, genetic factors, hypoxia, acidosis, and metabolic changes [290,291]. Changes in the peritumoral microenvironment related to pH changes and levels of neurotransmitters and their receptors are especially relevant for this review on ion channels. Peritumoral hypoxic zones within astrocytomas tend to be acidic, which leads to an increased inward Na^+^ current due to the activation of voltage-gated sodium channels lowering the seizure threshold [292]. The acidic TME may reduce the seizure threshold by additional mechanisms. Acidic pH inhibits inwardly rectifying potassium (Kir) channels reducing the resting membrane potential and thereby decreasing the seizure threshold. Acidosis also inhibits gamma-aminobutyric acid (GABA) conductance and disinhibits NMDA receptors, further increasing the likelihood of seizures (Figure 6A–C) [293].

Excitatory and inhibitory networks and their respective neurotransmitter systems also influence the risk of TAE (Figure 6A–C). Multiple lines of evidence support the loss of glutamate homeostasis as a factor in peritumoral seizure activity [126,294]. The known pathomechanism of glutamate provides a plausible basis for these observations [295]. Glutamate is produced by glioma cells as a byproduct of glutathione and is released in high amounts via the cystine-glutamate transporter (SXC). Its binding to NMDA and AMPA/Kainate receptors triggers the opening of cation-specific ion channels that permit the influx of Na^+^ or Ca^2+^ ions. Elevated extracellular glutamate concentrations in the peritumoral tissue of glioma patients promote the malignant behavior of glioma by exerting excitotoxicity of non-tumor parenchyma, thereby facilitating tumor expansion while also creating excitatory imbalance associated with a higher risk of seizures [296,297]. Overstimulation of NMDARs leads to excessive Ca^2+^ influx into neurons and excitotoxicity [125]. While the resulting neuronal cell death creates room for tumor expansion [126], the overactivation of AMPA receptors also mediates neural hyperexcitability and increases seizure risk (Figure 6B) [128].

Additional processes likely contribute to chronic hyperexcitability and TAE (Figure 6C) [126]. Studies in human glioma tissue, mouse and cell culture models indicate that a loss of GABAergic interneurons in peritumoral tissue combined with a reverse GABAergic effect from inhibitory to excitatory in surviving neurons creates a hyperexcitable TME with increased seizure susceptibility. In addition, downregulation of chloride exporter KCC2 and upregulation of chloride importer NKCC1 leads to excessive intracellular chloride concentrations and causes GABA-mediated depolarization and hyperexcitability [118,119,120,121,122].

#### 5.5.1. TME in Tumor-Associated Brain Edema (TABE)

Tumor-associated brain edema (TABE) is a common complication of brain tumors. In addition to physical compression of brain parenchyma by the tumor mass, cellular processes leading to BBB remodeling as discussed above play an important role. As described earlier, the movement of glioma cells along blood vessels leads to the displacement of astrocytic endfeet from endothelial cells, loss of endothelial tight junctions and break down of the BBB integrity. On a molecular level, this process is associated with the disorganization of the highly structured orthogonal arrays of particles (OAPs) related to AQP4 channels mentioned above.

#### 5.5.2. TME, Tumor pH, and Metabolic Environment

A metabolic hallmark of tumors is chronic extracellular metabolic acidosis resulting from hypoxia-independent aerobic glycolytic metabolism, which is referred to as the Warburg effect [298]. This altered metabolic tumor environment supports the metastatic behavior of tumor cells (discussed in Section 4, Glioma Metabolism). An acidotic TME promotes tumor progression and invasive behavior, which is positively correlated with lower pH [299]. Underlying mechanisms may include reduced tumor cell adherence, increased angiogenesis (discussed below), breakdown of the extracellular matrix, inhibition of anti-cancer immune response, and enhanced tumor cell migration [300,301,302,303].

To achieve and maintain a growth-permissive metabolic environment, tumor cells need to be able to sense protons. Sensors of the extracellular tumor tissue include acid-sensitive ion channels (ASICs, TRPs) or ion transporters (NHE1) and G protein-coupled H-sensors [298]. Ion channels can both sense pH and respond to pH change, either by directly modulating the membrane potential via control of ion flux or by activating intracellular signaling pathways. Ca^2+^ and Na^+^ ions are the most important ions in the context of tumor invasion and infiltration. Acid-sensing ion channels (ASICs) are H^+^, Ca^2+^, and Na^+^-gated cation channels that are activated by changes in the extracellular pH and may be involved in tumor proliferation and migration. Their role in pH-dependent proliferation and migration has been studied in various tumors [304]. Similarly, TRPs (transient receptor potential channels) are a family of pH-sensitive ion channels that may play a role in cancer [305]. Notably, high TRPM5 expression was correlated with poor overall survival in patients with different cancers [39]. The pH-dependent malignant behavior of tumor cells is also influenced by voltage-gated sodium channels (VGSC) [124]. Regulation of pH is one pathway the VGSC Na_v_1.5 (encoded by *SCN5A*) enhances infiltrative cancer behavior. It does so in a co-dependent manner with the Na^+^/H^+^ exchanger NHE1, which is an important regulator of H^+^ efflux. Na^+^ influx through NaV1.5 leads to increased H^+^ efflux through NHE1. The resultant NaV1.5/NHE1-dependent perimembrane acidification promotes pH-dependent extracellular matrix degradation and invasion (reviewed in [124]).

### 5.6. Angiogenesis and Blood Vessel Co-Option in Brain Tumors

In the brain, blood vessels contain two main cellular layers: the endothelial cells facing the blood (luminal) and the smooth muscles and pericytes facing the extravasal space in larger blood vessels and capillaries, respectively. As part of the highly structured blood–brain barrier (BBB), blood vessels participate in the tightly controlled barrier and in the molecular exchange between CNS and intravascular space. Glioblastoma (GBM), the most aggressive form of glioma is a highly vascular brain tumor. Within its vasculature are found two different types of vessels: those formed by angiogenesis (neoangiogenic vessels) and pre-existing vessels co-opted by tumor cells (co-opted vessels) [306].

#### 5.6.1. Angiogenesis in Brain Tumors

Angiogenesis is the growth of new capillaries from pre-existing blood vessels. Several angiogenic receptors and factors are upregulated in GBM and stimulate angiogenesis signaling pathways through activating oncogenes and/or downregulating tumor suppressor genes [307]. Neoangiogenic blood vessels play a central role in the progression of GBM. Physiological angiogenesis is a highly regulated process by which hypoxia triggers activating hypoxia-inducible factor-1 (HIF-1), a transcription factor that controls the expression of pro-angiogenic growth factors such as vascular endothelial growth factor (VEGF) [308], transforming growth factor-β (TGF-β) [309], fibroblast growth factors (FGFs) [310], angiopoietin-1 [311], epidermal growth factor (EGF) [312], matrix proteins [313], adhesion molecules [314], and metabolic proteins [315] to remodel the extracellular matrix components. In the pathologic angiogenesis of glioblastoma, many of the angiogenic pathways and factors known from physiological angiogenesis, such as VEGF, FGFs, TGF-β, MMPs, and angiopoietins, as well as hepatocyte growth factor (HGF) and platelet-derived growth factor (PDGF), are upregulated by the hypoxic tumor environment and as a result of tumor suppressor loss. The hypoxic TME induces the differentiation of cancer stem cells to endothelial progenitor cells and mature endothelium. The newly created neoangiogenic tumor vessels are functionally immature, dysfunctional, and hyperpermeable, which further contributes to a chronic hypoxic and nutrient-deprived state promoting tumor progression. Abnormal tumor neoangiogenesis also contributes to treatment resistance [316,317].

#### 5.6.2. Co-Option in Brain Tumors

Co-option is a non-angiogenic process whereby GBM cells directly utilize the pre-existing vasculature of the non-malignant tissue as a supply of oxygen and nutrients to promote invasion and infiltration [318]. Blood vessel co-option involves the process of glioma cells moving along pre-existing vessels within the extracellular space and displacing astrocytic endfeet from endothelial or mural cells. The disruption increases BBB permeability, extravasation of fluid, and disintegration of the gliovascular unit, ultimately facilitating peritumoral brain edema. Vessel co-option is an underappreciated mechanism of tumor vascularization that can influence disease progression, infiltration, and response to treatment. It has been demonstrated as a mechanism of acquired resistance to antiangiogenic therapy in several tumor models [105].

#### 5.6.3. TRPC6 in Angiogenesis of Glioma

Transient receptor potential (TRP) channels, as mentioned earlier, belong to a superfamily of nonselective cation channels involved in sensing and responding to changes in the TME related to glioma. In addition to having important functions in the pathophysiology of GBM, TRP channels have been studied for their potential as pharmacological targets and biological markers. Subtypes of this channel family have been discussed in previous sections in the discussion of the BBB and the TME. Several TRP channels have been studied as potential biological markers of glioma progression and prognosis, including TRPC1, TRPC6, TRPM2, TRPM3, TRPM7, TRPM8, TRPV1/2. As previously mentioned, their expression has been positively correlated with the overall survival of glioma patients [319].

TRPC6 is the only TRP channel shown to be involved in glioma angiogenesis [91]. While TRPC6 is thought to be important for VEGF-mediated angiogenesis [90], in GBM, this was demonstrated to be a hypoxia-induced Notch1-dependent process [14]. Notch is a well-described regulator of cell fate proliferation and migration during the normal development of many tissues and cell types. Inhibition of the Notch pathway in GBM blocked hypoxia-induced upregulation TRPC6. The upregulation of TRPC6 is thought to promote angiogenesis in GBM cells via the TRPC6-calcineurin-NFAT pathway. This is supported by the observation that inhibition of hypoxia-induced TRPC6 expression and NFAT activation markedly reduces the number of branch points in vitro using hypoxia-induced endothelial cell tube formation. In clinical biopsies, expression of Notch and TRPC6 was elevated in GBM biopsies in comparison with normal brain tissues.

#### 5.6.4. Other TRP Channels in Tumor Angiogenesis

Several TRP channels previously reported to be involved in various aspects of tumorigenesis are also expressed in the brain vasculature (TRPM2, TRPM7, TRPV2, TRPV4), but their specific role in GBM angiogenesis has not been elucidated [104,320,321,322]. Studies in the Lewis lung carcinoma tumor model of TRPV4 (ECKO) mice demonstrated that endothelial TRPV4 is a critical modulator of vascular integrity and tumor angiogenesis and deletion of TRPV4 promoted tumor angiogenesis, growth, and infiltration [323]. In several cell lines, including uveal melanoma, endothelial, and corneal epithelial cells, activation of TRPM8 inhibited the VEGF transactivation of TRPV1 and the consequent pro-tumorigenic effects mediated by the VEGFR. Furthermore, TRPV1 was upregulated in uveal melanoma cells suggesting that TRPM8 is a potential drug target for suppressing VEGF-induced increases in neovascularization and uveal melanoma growth. The crosstalk found between TRPM8, TRPV1, and VEGF also points to potential interactions with other TRP channels, other channel families such as BK channels [99], and the G protein-coupled receptor (GPCR) genes [324], in the modulation of signaling pathways that determine fundamental aspects of tumor biology including angiogenesis. These findings also provide a window into possible developments of targeted TRP-based antiangiogenic pharmacotherapies, which are gaining traction given the availability of novel formulations [91,325,326,327].

## 6. Therapeutic Outlook for Ion Channels in Brain Cancer

Therapeutic approaches to brain tumors vary with location, type, and extent of tumor progression. For the most aggressive forms, such as glioblastoma (GBM), the current standard of care includes surgical resection, radiotherapy, and chemotherapy, but despite improvement of multimodal regimens, the prognosis remains poor with a median survival for GBM patients of 12–15 months after diagnosis and a 5-year survival rate of less than 5% [328]. Some of the characteristics of malignant brain tumors and particularly of GBM, also provide the basis for the resistance to standard treatment. As outlined in this review, these include the BBB, the extensive tumor heterogeneity, and various mechanisms of acquired treatment resistance in tumor cells.

Here, we provide a spotlight on current research for novel therapeutic avenues with a focus on strategies to exploit the properties of ion transporters across membranes to modulate tumor behavior or treat tumor-associated epilepsy (TAE). Ion channels are essential for the survival of normal and tumor cells, and mounting evidence supports their role in the progression from normal to malignant state [329]. Several lines of research provide compelling support for pharmacological targeting of a wide range of ion channels, including Ca^2+^, Na^+^, and K^+^ channels, anion channels, as well as water channels, to modify tumor behavior and to treat tumor-related complications such as peritumoral edema and tumor-associated epilepsy [330]. However, despite promising premises, only a few concepts that capitalize on manipulating ion channel function to influence the growth, progression, and outcomes of brain tumors have progressed to clinical trials.

### 6.1. Repurposing of Drugs

Ion channel modulators are a mainstay in the treatment of tumor-associated epilepsy (TAE), which has prompted attempts to use these agents, many of them routinely used to treat neurological complications, as enhancing adjuvants of antineoplastic therapy. Multiple repurposing strategies for antiepileptic drugs (AEDs) have yielded interesting results. Among those are sodium channel blockers and modulators (carbamazepin, phenytoin, lamotrigine, lacosamide, topiramate), calcium channel modulators (gabapentin, pregabalin), AMPA receptor antagonists (perampanel, topiramate), GABA receptor enhancers (phenobarbital, valproate, topiramate), and others. Levetiracetam, in addition to inhibiting synaptic vesicle protein SV2A, also exerts a partial blockade of N-type calcium currents [293].

Phenytoin and carbamazepine are antiepileptic drugs (AEDs) that block voltage-gated sodium channels (VGSC). They also have been shown to inhibit proliferation and growth in various cancers and have been considered for the treatment of gliomas [331,332]. Valproate works as a GABA receptor enhancer, Na^+^ channel blocker, and Ca^2+^ channel blocker and previously reached phase III in clinical trials in the treatment of glioma. Valproate also functions as a histone deacetylase inhibitor (HDAC). In a recent study, combining valproate with the anti-cancer drug temozolomide (TMZ) provided a survival benefit to patients with wild type p53 glioblastoma but not mutated p53 [333].

A different clinical class of pharmaceuticals is Tamoxifen, a selective estrogen receptor modulator (SERM) that blocks estrogen action by binding to the estrogen receptor (ER) in breast cancer and has been widely used during the last 30 years for the treatment of breast cancer [334]. It also acts as a multichannel blocker that inhibits several K^+^ channels [335] and may also exert a chemotherapeutic effect on high-grade glioma, which was demonstrated on TMZ-resistant glioblastoma cell lines U251 and BT325 [336].

A group of drugs that is perhaps best known as antidiabetics, the biguanides, exert inhibitory function on Cl^−^ channels. Metformin, a type 2 diabetic agent [337], has been linked to improved outcomes in patients with various cancers [338]. In cell culture, it appears to have multiple effects alone or in synergistic combination with TMZ, including inhibition of proliferation and expansion of glioblastoma stem cells, and decreasing acquired resistance of GBM cells to TMZ [339,340,341,342]. Metformin also promotes the differentiation of glioma-initiating cells into non-tumorigenic cells via FOX03 activation [343]. Larger studies reporting clinical outcomes are mixed for metformin, which may, in part, be due to different study designs. Metformin monotherapy was among the most important predictors for survival in a retrospective analysis on 988 patients [344]. In contrast, a recent pooled analysis of three prospective randomized clinical trials found no association between metformin and survival [345].

### 6.2. Potential Pharmacological and Nonpharmacological Targets

As discussed in previous sections, the transient receptor potential (TRP) ion channels are a superfamily of cation-transporters with calcium permeability involved in intracellular Ca^2+^ homeostasis that contribute to the malignant behavior of glioblastoma. Here, we highlight some of the recent efforts to harness insights into their biology to identify novel therapeutic targets for the clinical therapy of gliomas.

One example is TRP vanilloid-2 (TRPV2), for which increased expression has been associated with temozolomide-resistant glioblastoma, tumor recurrence, and decreased patient survival [346]. Conversely, TRPV2 has been shown to inhibit glioblastoma cell proliferation and overcome their resistance to carmustine. This effect can be activated by cannabidiol (CBD), a relatively non-psychoactive cannabinoid that appears to act as a selective TRPV2 agonist in glioma cell lines [347]. Cannabinoids exert their effects primarily through CB1 and CB2 receptors. In addition, cannabinoids can modulate a certain subset of TRP channels, including TRPV1, TRPV2, TRPV3, TRPV4, TRPA1, and TRPM8. CBD has been found to be the most potent and efficacious phytocannabinoid that activates TRPV2 [348]. In vitro, activation of TRPV2 through CBD has been shown to induce GSC differentiation and GSC autophagy, inhibit GCS proliferation and clonic expansion, and overcome their resistance to carmustine [349]. Several clinical trials have investigated a related cannabinoid, delta(9)-tetrahydrocannabinol (Δ9-THC), for the treatment of glioblastoma (reviewed in [350]). The only placebo-controlled clinical trial of 21 patients with glioblastoma using CBD in a mixture with Δ9-THC (nabiximol) combined with temozolomide was industry-sponsored (Jazz Pharmaceuticals, formerly GW Pharmaceuticals). The randomized portion of this trial which was designed as a phase 1b safety and tolerability study included 12 patients in the treatment arm, and 9 patients in the placebo group. Due to several limitations including the limited number of enrolled patients, a high drop-out rate, lack of prespecified power calculation, and lack of efficacy endpoints, the results of this study did not reach significance and are difficult to interpret [351]. [Ref. Twelves, 2021 #1435]. Other clinical experience is limited to small case studies [352,353].

TRP melastatin-7 (TRPM7) is a non-specific divalent cation channel coupled with an atypical serine/threonine-protein kinase domain at its C-terminus. Overexpression of TRPM7 has been observed in several cancers and other disease states [354], and both the ion channel properties, as well as the kinase domain, are suspected of contributing through multimodal mechanisms [355,356]. Emerging data point to a potential value of TRPM7 as a biomarker and therapeutic target in cancer [355]. Several small organic molecules have been identified that either activate or inhibit the TRPM7 channel moiety or regulate its kinase activity, which holds promise for the development of drugs targeting TRPM7 (reviewed by [357]). Among TRPM7 modulating agents, carvacrol was identified as a novel inhibitor of *Drosophila* TRPL and their homologous mammalian TRPM7 channels [358]. Subsequently, TRPM7 was found to be upregulated in glioblastoma, and inhibition of TRPM7 reduced GBM proliferation, migration, and invasion [359]. Very recently, inhibition of TRPM7 by carvacrol in a xenograft GBM mouse model led to reduced tumor size in mice injected with U87MG and U251 cells. This was associated with decreased p-Akt protein levels and increased p-GSK3β protein levels [360]. The PI3K/Akt signaling pathway promotes GBM progression, and its activation has been associated with treatment resistance and poor prognosis in GBM patients; GSK3-β is a component of the PI3K/Akt signaling pathway [361]. While the data provide a promising outlook, clinical studies have been scarce in this field, and there is still much research to be performed on the therapeutic potential of TRP channels for GBM treatment.

Ion channels have also been proposed to mediate non-pharmacological treatment options of glioblastoma. Electrotherapy in combination with chemotherapeutics may provide clinical benefit. One modality of electrotherapy approved in the United States and Europe for treatment of primary and recurrent adult glioblastoma generates alternating electric fields delivered directly to the patient onto defined tumor treating fields (TTF) [328]. A phase III clinical trial documented significant improvement in both overall and progression free survival when electrotherapy was used in combination with maintenance Temozolomide (TMZ) when compared with TMZ alone [362] [Ref]. Electrotherapy via TTF appears to inhibit mitosis by interfering with the formation of the spindle apparatus through targeting tubulin polymerization [363] [Ref]. Although some authors have suggested ion channels to be key in understanding the antineoplastic effects of TTF [328], this hypothesis has not been systematically verified experimentally and further studies are needed to better understand their involvement in this emerging field of translational research.

## 7. Conclusions and Future Directions

In recent years, ion channels have moved into the spotlight of brain cancer. Accumulating research describes their multifaceted roles in cancer hallmarks and tumor contexts, such as histology, genetic background, cell type, metabolism, malignant behavior and metastasis, microenvironment, and angiogenesis. While these factors all influence prognosis and treatment response, ion channels have become potential treatment targets in their own right. A critical obstacle to the successful modification of ion channel behavior toward the desired outcome in the treatment of brain cancer is the sheer complexity that governs ion channel function in the heterogeneous tumor microenvironment. As single-cell sequencing becomes more accessible, we will increasingly achieve better resolution of the cell types that make up heterogenous gliomas. This is expected to improve the categorization of tumor subtypes and help determine on a cellular and microenvironmental level which ion channel behaviors are part of the intrinsic tumor program, and which are responses to the tumor environmental changes. Such high-resolution molecular data are needed on a large scale to identify those pharmacological ion channel targets that hold promise to progress to clinical trials. The scarcity of successful clinical trials despite a plethora of potential targets at the crossroads of ion channels and brain cancer is likely at least in part a reflection of our incomplete understanding of “real world” ion channel biology. Further efforts toward larger clinical trials that include clinical specimens may hold promise for more tailored and effective treatments. Such approaches will also be helpful in revealing the transcriptional changes that occur upon metastatic transformation and after treatment. By better understanding the molecular makeup of tumor cells, one can also achieve more insight into the genes that drive tumor behaviors. What are the hallmarks of glioma stem cells? If we can target them, can we limit recurrence?

To put it in another way, sequencing data will help us define whether there truly are ion channel hallmarks that define aspects of glioma and tumor behavior. Do proliferating cells generally utilize the same ion channels for their behavior or do oncogenic programs alter ion channel profiles to permit uncontrolled growth? Ion channels are highly druggable—if we can better understand their cell type-specific behavior and their roles in cancer, great promise awaits more effectively treating troublesome tumors.

## Figures and Tables

**Table 1:** Ion channel expression correlation with glioma grade and patient survival. Studies examining patient glioblastoma samples (GBM), patient-derived cell lines, and glioma cell lines have found correlations with specific ion channel expression, suggesting that ion channels may serve as biomarkers. N/A, not applicable.

Ion Channel	Expression	Model	Patient Survival	Reference
EAG	Increased	GBM metastases	Expression correlated with worse survival	[9]
hERG	Increased	GBM-derived cells	Expression correlated with worse survival	[10]
BK	Increased	GBM-derived cells, GBM cell lines U251, U87MG	Expression correlated with malignancy	[11]
TRPV1	Decreased with glioma grade	Normal human astrocytes, U87MG, U737 glioma cell lines	Expression promotes patient survival	[12]
TRPV2	Decreased with glioma grade	U97MG, primary glioma cells	Expression correlated with glioma grade	[13]
TRPC6	Increased	GBM patient samples	N/A	[14]
TRPC1	Increased	Patient glioma samples	Expression positively correlated with patient survival	[12]
TRPM2	Increased	Patient glioma samples	Expression positively correlated with patient survival	[12]
TRMPM3	Increased	Patient glioma samples	Expression positively correlated with patient survival	[12]
TRPM7	Increased	Patient glioma samples	Expression positively correlated with patient survival	[12]
TRPM8	Increased	Patient glioma samples	Expression positively correlated with patient survival	[12]
VRAC	Increased	GBM cell lines	N/A	[15,16,17]
TMEM16A	Increased	Patient GBM samples, GBM cell lines: U87MG, U118, U251, SHG44	Increased with grade, less favorable to patient survival	[18,19]
CLIC1	Increased	GBM	Expression correlated with worse prognosis	[20]
NHE5	Increased	C6 glioma cells	N/A	[21]
ASIC1	Decreased	GBM samples	Expression negatively correlated to survival	[22]
PIEZO	Increased	Human GBM	Expression inversely correlated to survival	[23,24]
NKCC1	Increased	Primary GBM samples	Expression positively correlated with grade	[25,26]
IK	Increased	Patient GBM samples	Expression negatively associated with survival	[27]
NHE1	Increased	Primary human glioma cells	N/A	[28,29,30]
Kir4.1	Decreased or mislocalized	Glioma cell lines	N/A	[31,32]
mitoBKCa	Increased	Glioma cell line LN229	N/A	[33]
AQP1	Increased	Astrocytomas, GBM	N/A	[34,35,36]
AQP4	Mislocalized	Patient GBM samples	N/A	[37,38]
TRPM5	Increased	Patient cells	Expression negatively associated with survival	[39]

**Table 2:** Ion channel behaviors. This table summarizes cell behaviors of various ion channels: proliferation (clear), metastasis and invasion (yellow), metabolism (red), angiogenesis and tumor microenvironment (TME) (blue), apoptosis, or seizure-related behavior (green). BBB, blood–brain barrier. “↑” indicates increased, “↓” represents decreased, “ →” indicates, for example in G1 → S, cell cycle progression from G1 to S phase. *GABR*, Gamma-aminobutyric acid type A receptor subunits (multiple).

Ion	GENE	Channel Name(s)	Cell Behavior	Reference
K^+^	*KCNJ10*	Kir4.1	↓Proliferation	[48]
			↓Fillopodia and invasion	[32]
			Endothelial BBB	[49,50,51,52,53,54,55,56,57]
K^+^	*KCNH2*	Kv11.1, hERG	↑Proliferation, G1→S	[10,58,59]
K^+^	*KCNJ11*	KATP, Kir6.2	↑Proliferation, G1→S	[60]
K^+^	*KCNMA1*	KCa1.1, BK, mitoBKCa (splice variant)	↑Proliferation, S→G2	[61]
			↑Migration, swelling at leading edge	[62,63,64,65,66]
			MitoBK promotes apoptosis	[33]
K^+^	*KCNN4*	KCa3.1, IK	Irradiation, ↑Proliferation, G2→M	[27]
			↑Migration and invasion, trailing edge	[16,17,27,67,68,69,70,71,72]
K^+^	*KCNA1*	KV1.1, Shaker	Endothelial BBB	[49,50,51,52,53,54,55,56,57]
K^+^	*KCNA3*	KV1.3, mitoKV1.3	MitoKV1.3 promotes apoptosis	[73]
K^+^	*KCNJ8*	(KIR) Kir6.1	Pericyte BBB expression	[74,75,76,77,78,79]
Cl^−^	*CLIC1*	CLIC1	↑Proliferation, G1→S	[80]
Cl^−^	*CLCN3*	CLC3	Premitotic condensation, cell cycle speed	[81,82]
			↑Migration and invasion, ↑MMP—Extracellular matrix (ECM) remodeling	[69,70,83,84]
Cl^−^	*LRRC8A*	VRAC, IClswell1	Hypoxia-related proliferation and survival	[85]
Cl^−^	*TMEM16A*	TMEM16A, ANO1	↑Proliferation, ↑Growth factor	[18,19]
Cation	*TRPC1*	TRPC1	↑Proliferation, G2→M	[86]
			↑Directional migration, leading edge	[87]
TRPC6	*TRPC6*		↑Proliferation, ↑Growth factor	[14,88,89]
			↑Hypoxic migration	[14]
			↑VEGF-mediated angiogenesis	[90,91]
Cation	*TRPM2*	TRPM2	↑Apoptosis	[92,93]
Cation	*TRPM7*	TRPM7	↑Proliferation	[94,95,96]
			↑Migration and invasion through kinase domain	[94]
Cation	*TRPM8*	TRPM8	↑Proliferation, S→G2	[97,98,99]
			↑Migration mediated by BK activation	[97,100]
Cation	*TRPML1*	MCOLN1	↑Apoptosis, ROS sensor?	[101]
Cation	*TRPML2*	MCOLN2	↑Proliferation, G0→G1, ↓Apoptosis	[102,103]
Cation	*TRPV1*	TRPV1	↓Proliferation	[12]
Cation	*TRPV2*	TRPV2	↓Proliferation	[13]
Cation	*TRPV4*	TRPV4	↑Migration and invasion, ↑Cytoskeletal remodeling	[104]
			↑Angiogenesis	[105]
Na^+^	*ASIC1*	ASIC1	↓Proliferation	[22,106]
			↑Migration and lamellipodium expansion	[107,108,109,110]
Na^+^/Ca^2+^/K^+^	*PIEZO1*	PIEZO1	↑Proliferation, tissue stiffness	[23]
			↑Focal adhesion formation	[23]
Open: ADP/ATP, Cl^−^, NADH/NAD^+^, Closed: Ca^2+^	*VDAC1*	VDAC1	↑Oxidative phosphorylation, translates cellular state to mitochondria function, can drive apoptosis	[111,112,113]
	*VDAC2*	VDAC2	Negatively regulates glycolysis via PFK direct interaction	[114]
Cl^−^, HCO_3_^−^	*SLC4A3*	Cl^−^/HCO_3_^−^ exchanger	Pericyte BBB expression	[74,75,76,77,78,79]
Cl^−^, HCO_3_^−^	*SLC4A4*	Cl^−^/HCO_3_^−^ exchanger	Pericyte BBB expression	[74,75,76,77,78,79]
Na^+^/I^−^	*SLC5A5*	Na^+^/I^−^ symporter	Pericyte BBB expression	[74,75,76,77,78,79]
Na^+^/Ca^2+^	*SLC8A2*	Na^+^/Ca^2+^ exchanger	Pericyte BBB expression	[74,75,76,77,78,79]
Na^+^/Ca^2+^	*SLC8A3*	Na^+^/Ca^2+^ exchanger,	Pericyte BBB endothelial cells	[49,50,51,52,53,54,55,56,57]
Na^+^/H^+^	*SLC9A1*	NHE1	↑Glycolysis	[29,30,115]
Na^+^/H^+^	*SLC9A3R1*	NHE 3R1	Pericyte BBB expression	[74,75,76,77,78,79]
Na^+^/H^+^	*SLC9A5*	NHE5	↑Proliferation	[21]
			↑Metastasis, cell adhesion, invasion	[21]
Na^+^/H^+^	*SLC9A9*	NHE9	↑Proliferation, ↑Growth factor signaling	[116,117]
Na^+^/K^+^/Cl^−^	*SLC12A2*	NKCC1	↑Migration, invasion, filipodia formation, and focal adhesions	[25,26]
			Expressed in BBB endothelial cells, role downregulated in GABAergic hyperexcitability	[49,50,51,52,53,54,55,56,57]
K^+^, Cl^−^	*SLC12A5*	KCC2	Downregulated contributes to GABA-mediated hyperexcitability in peritumoral tissue	[118,119,120,121,122]
H^+^	*SLC15A2*	H(+)-coupled peptide transporter	Pericyte BBB expression	[74,75,76,77,78,79]
Na^+^/K^+^/Ca^2+^	*SLC24A3*	Na^+^/K^+^/Ca^2+^ exchanger	Pericyte BBB expression	[74,75,76,77,78,79]
Na^+^/K^+^	*ATP1A1*	Na^+^-K^+^-ATPase	Expressed in BBB endothelial cells, expressed in BBB pericytes	[49,50,51,52,53,54,55,56,57]
Ca^2+^, Na^+^	*ATP2B2*	Ca^2+^-ATPases	Pericyte BBB expression	[74,75,76,77,78,79]
K^+^	*ABCC9*	ATP-sensitive K^+^ channel, ATP-binding cassette	Pericyte BBB expression	[74,75,76,77,78,79]
H_2_O	*AQP1*	Aquaporin 1	↑Migration	[123]
			↑Angiogenesis	[34,35,36]
H_2_O	*AQP4*	Aquaporin 4	Contribute to BBB remodeling and brain edema in glioma	[37,38]
Na^+^	*SCN5A*	NaV1.5	↑Invasion with NHE1	[124]
Ca^2+^, Na^+^	*GLUN*	NMDAR	Overstimulation of NMDARs leads to excessive Ca^2+^ excitoxicity, cell death of TME provides room for tumor expansion	[125,126]
Na^+^	*GLUA*	AMPAR	↑Proliferation	[42,127]
			↑Invasion	[42,127]
			Hyperexcitable/seizure risk	[128]
Cl^−^	*GABR*	GABA	neurons die and neurotransmitter shifts polarity, causing hyperexcitability	[118,119,120,121,122]

## Data Availability

Not applicable.
